# Mechanism‐Driven Nanoformulations for Cognitive Impairment Induced by Intermittent Hypoxia and Periodontitis

**DOI:** 10.1002/adma.73920

**Published:** 2026-07-07

**Authors:** Kai‐Run Zhang, Tianbao Zhu, An‐Qi Liu, Jia‐Jia Deng, Ya‐Li Han, Sheng‐Cai Qi, Xue Liu, Tian‐Yi Jiang, Ming‐Rui Zhai, Yu‐Ying He, Xiu‐Juan Li, Hong‐Ting Da, Rui Xu, Xiaomin Li, Jun Gao, Yue‐Hua Liu

**Affiliations:** ^1^ Department of Orthodontics, Shanghai Stomatological Hospital & School of Stomatology, Shanghai Key Laboratory of Craniomaxillofacial Development and Diseases Fudan University Shanghai China; ^2^ Laboratory of Advanced Materials, Department of Chemistry, Shanghai Key Lab of Molecular Catalysis and Innovative Materials and State Key Laboratory of Molecular Engineering of Polymers, College of Chemistry and Materials Fudan University Shanghai China; ^3^ Shanghai Key Laboratory of Craniomaxillofacial Development and Diseases, Shanghai Stomatological Hospital & School of Stomatology Fudan University Shanghai China; ^4^ Department of Orthodontics, Shanghai Ninth People's Hospital, College of Stomatology, Shanghai Jiao Tong University, School of Medicine. National Clinical Research Center For Oral Diseases Shanghai Key Laboratory of Stomatology & Shanghai Research Institute of Stomatology Shanghai China; ^5^ Department of Prothodontics, Shanghai Stomatological Hospital & School of Stomatology, Shanghai Key Laboratory of Craniomaxillofacial Development and Diseases Fudan University Shanghai China; ^6^ Department of Multidisciplinary Consultant Center, Shanghai Key Laboratory of Craniomaxillofacial Development and Diseases, Shanghai Stomatological Hospital & School of Stomatology Fudan University Shanghai China; ^7^ Department of Neurobiology, School of Basic Medical Science Nanjing Medical University Nanjing China; ^8^ Department of Laboratory the First Affiliated Hospital of Anhui Medical University Hefei China; ^9^ College & Hospital of Stomatology, Anhui Medical University Anhui Province Key Laboratory of Oral Diseases Research Hefei China; ^10^ Stomatological Institute of Integrated Innovation, Shanghai Stomatological Hospital & School of Stomatology Fudan University Shanghai China

**Keywords:** cognitive impairment, intermittent hypoxia, nanoformulations, oxidative stress, periodontitis

## Abstract

Cognitive impairment is a growing global health challenge, yet its multifactorial drivers remain incompletely understood. Intermittent hypoxia (IH) and periodontitis are identified as significant independent risk factors that frequently co‐occur. This study investigates the mechanisms through which these conditions accelerate cognitive decline. Here, utilizing a comorbid mouse model, we demonstrate that outer membrane vesicles (OMVs) derived from *Porphyromonas gingivalis* exacerbate IH‐induced mitochondrial oxidative stress and neuroinflammation, leading to neuronal damage and mitochondrial dysfunction in the hippocampus. Specifically, this process is driven by the activation of the HIF‐1α/HMGB1/NLRP3 signaling axis. To target this pathology, an asymmetric RVG‐Au&mSiO_2_‐TPP‐VB Janus nanoformulation is developed for hierarchical neuron‐to‐mitochondria targeting. The inherent asymmetry of the Au&mSiO_2_ Janus architecture enables anisotropic, spatially segregated functionalization of its two distinct domains. The neuron‐targeting RVG peptide and the mitochondria‐targeting ligand TPP are anchored on distinct domains, while vitamin B2 (VB) is encapsulated within the mesopores. Intranasal administration of this nanoformulation achieves precise mitochondrial accumulation in neurons, effectively scavenging reactive oxygen species, suppressing neuroinflammation, and significantly restoring cognitive function. Our findings reveal a molecular pathway through which IH and periodontitis jointly drive cognitive dysfunction and propose a hierarchically targeted nanotherapy for related comorbidities.

## Graphical abstract

Clinically, intermittent hypoxia (IH) is often accompanied by periodontitis, which accelerates cognitive impairment induced by IH. Novel Janus mesoporous gold‐silica nanoformulations (RVG‐Au&mSiO_2_‐TPP‐VB) enable intranasal delivery, achieve hierarchical targeting to neuronal mitochondria, effectively scavenge reactive oxygen species, inhibit the HIF‐1α/HMGB1/NLRP3 axis, alleviate neuroinflammation, and can significantly restore cognitive impairment caused by IH and periodontitis.

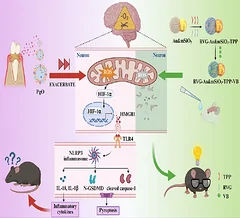

## 1 Introduction

Homeostasis of the central nervous system (CNS) is essential for normal cognitive function but is highly vulnerable to pathological intermittent hypoxia (IH) [1, 2], a hallmark of disorders such as obstructive sleep apnea [3, 4], chronic obstructive pulmonary disease [5], and congestive heart failure [6]. Unlike sustained hypoxia, IH is characterized by cyclical oxygen fluctuations, which trigger oxidative stress, immune activation, and metabolic dysregulation, thereby contributing to cognitive impairment [7, 8]. Consistent with this, patients with IH‐related disorders frequently exhibit deficits in learning, memory, and executive function [9, 10, 11, 12, 13]. However, the factors that modulate individual susceptibility to IH‐induced cognitive dysfunction remain incompletely understood.

Periodontitis is a prevalent chronic inflammatory disease driven by dysbiotic oral microbiota and is increasingly recognized as a systemic risk factor beyond the oral cavity. Epidemiological studies report a high prevalence of periodontitis in patients with IH‐related disorders, suggesting a potential interaction between chronic peripheral infection and hypoxia‐driven pathology [14, 15]. Among periodontal pathogens, *Porphyromonas gingivalis* (*Pg*) has been identified as a keystone species capable of evading host immune clearance and disseminating systemically. Its virulence factors, including outer membrane vesicles (PgO), can enter the bloodstream, disseminate to distant organs, and trigger neuroinflammatory responses. Notably, *Pg* components have been detected in the brains of patients with cognitive decline, and epidemiological evidence links periodontitis to an increased risk of Alzheimer's disease (AD) [16, 17]. However, whether PgO directly modulates IH‐induced neural injury and cognitive impairment remains unclear.

A key mechanism underlying IH‐related neural damage is the dysregulation of mitochondrial metabolism and excessive production of reactive oxygen species (ROS) [18]. These processes are closely modulated by hypoxia‐inducible factor‐1α (HIF‐1α), a transcription factor that orchestrates cellular responses to fluctuating oxygen availability. Beyond metabolic regulation, HIF‐1α can promote the release of high‐mobility group box 1 (HMGB1) [19]. Under pathological conditions, HMGB1 acts as a damage‐associated molecular pattern (DAMP), promoting innate immune activation via receptors such as TLR4 [20]. Importantly, both IH and periodontitis have been independently shown to activate the nucleotide‐binding oligomerization domain‐like receptor 3 (NLRP3) inflammasome, a key driver of caspase‐1 activation and inflammatory pyroptosis [21, 22, 23]. However, whether these pathways converge under conditions of combined IH and periodontal infection, and how such convergence contributes to neuronal injury, remains unresolved.

**FIGURE 1:**
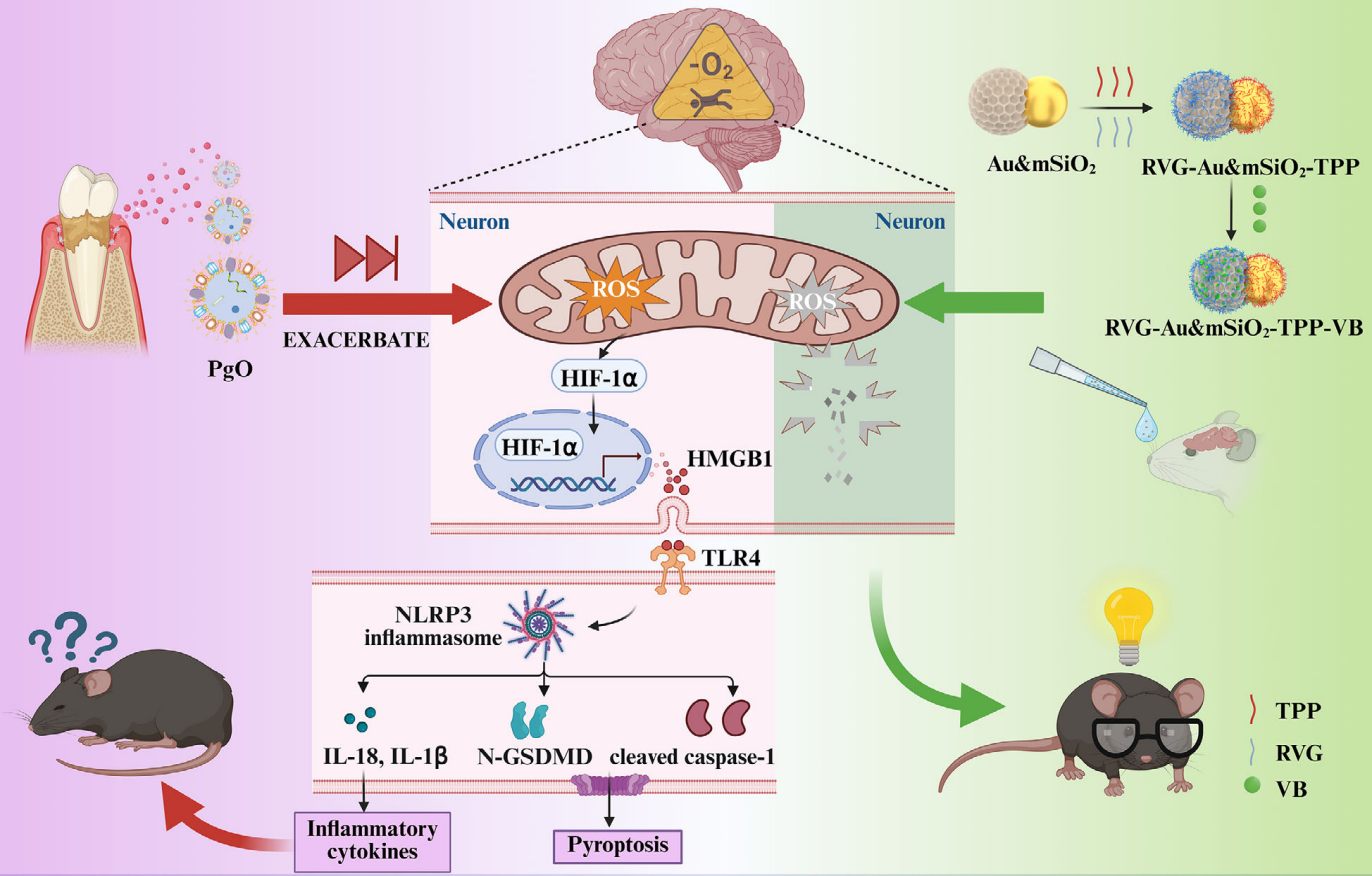
The schematic illustration of PgO‐aggravated IH‐induced cognitive impairment and its mitigation with a hierarchical neuron‐to‐mitochondria targeted Janus nanoformulation. Periodontal pathogen‐derived PgO amplifies mitochondrial ROS production under IH conditions, leading to activation of the HIF‐1α/HMGB1/NLRP3 signaling cascade and subsequent neuronal pyroptosis. Leveraging the intrinsic asymmetry of the Au&mSiO_2_ Janus architecture, neuron‐targeting RVG peptide and mitochondria‐homing TPP are spatially segregated on opposite domains, while VB is encapsulated within the mesoporous mSiO_2_ compartment. This hierarchical targeting strategy enables precise delivery of VB to neuronal mitochondria, thereby alleviating oxidative stress, suppressing neuroinflammation, and ultimately ameliorating IH‐associated cognitive deficits.

In this study, we aimed to elucidate the molecular mechanisms by which periodontal pathogen‐derived outer membrane vesicles exacerbate IH‐induced cognitive impairment, while simultaneously exploring a hierarchical targeting therapeutic strategy to counteract this pathology. As shown in Figure 1, we used a comorbid mouse model integrating IH exposure and periodontitis, and combined behavioral assessments, functional magnetic resonance imaging (fMRI), and molecular assays to investigate the role of the HIF‐1α/HMGB1/NLRP3 axis in mediating hippocampal neuronal injury under conditions of combined hypoxia and infection. To further address this mechanism‐driven therapeutic need, we engineered an asymmetric RVG‐Au&mSiO_2_‐TPP‐VB Janus nanoformulation designed for hierarchical neuron‐to‐mitochondria targeting [24, 25]. In this platform, the neuron‐targeting rabies virus glycoprotein‐29 peptide (RVG) is selectively conjugated to the Au domain, while the mitochondria‐homing moiety triphenylphosphonium (TPP) is anchored on the mSiO_2_ compartment, within whose mesoporous channels the antioxidant vitamin B2 (VB) is encapsulated [26, 27, 28, 29, 30]. Intranasal administration of this nanoformulation achieved precise mitochondrial accumulation [31, 32], effectively scavenged ROS, suppressed neuroinflammation, preserved mitochondrial integrity, and significantly restored cognitive function.

## 2 Results

### 2.1 PgO Accelerated IH‐Induced Cognitive Dysfunction

**FIGURE 2:**
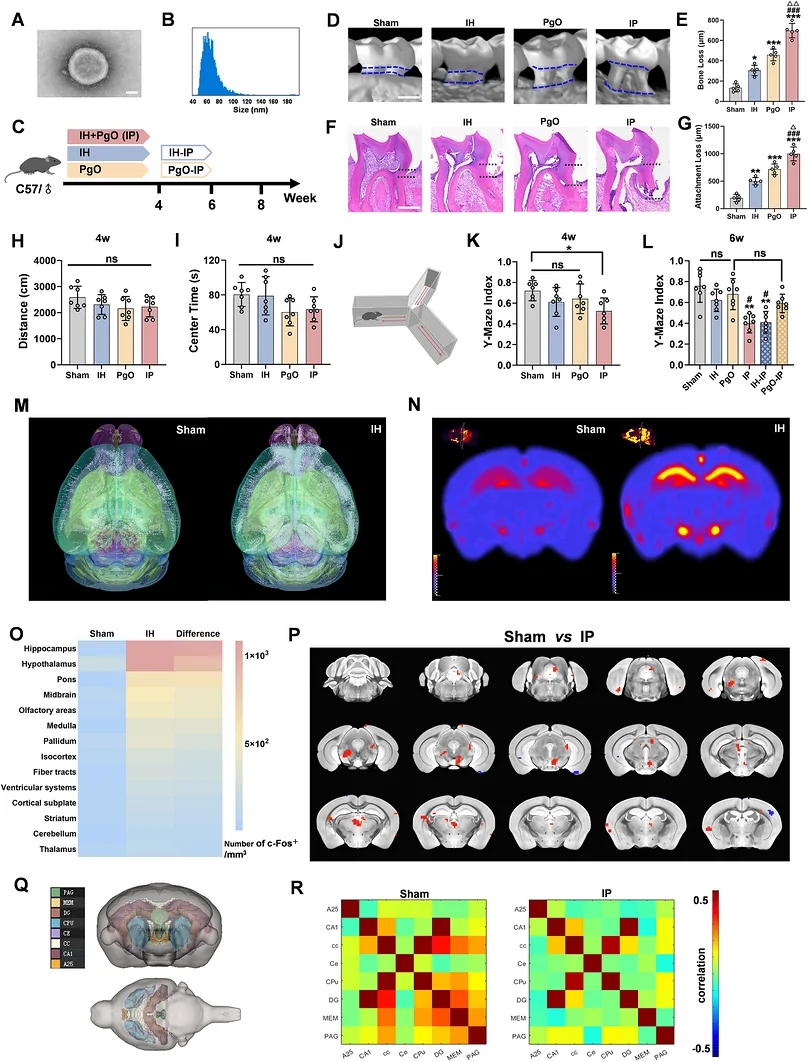
PgO acts as a pathological accelerator that markedly hastens the onset and progression of IH‐induced cognitive decline. (A) TEM and (B) NTA characterization of PgO (scale bar: 20 nm). (C) The schematic diagram of the experimental timeline for mouse model establishment. (D,E) Micro‐CT and (F,G) H&E staining of mouse alveolar bone (*n* = 5; scale bar: 500 µm), after 4 weeks post‐modeling; (H,I) locomotor activity in the open‐field test; (J) Schematic illustration of Y‐maze apparatus; (K,L) Spontaneous alternation performance in the Y‐maze test (*n* = 7). (M) c‐Fos expression in different brain regions of Sham and IH mice after tissue clearing and imaging; (N) Heat map of c‐Fos expression in the hippocampal region; (O) Heat map of c‐Fos expression across different brain regions (*n* = 3). (P) Seed‐based functional connectivity analysis of CA1 using resting‐state fMRI data. Voxels with significant between‐group differences (Sham vs. IP) are projected onto standard brain templates. Red: stronger connectivity in the Sham group relative to the IP group. Blue: weaker connectivity in the Sham group relative to the IP group. (Q) Three‐dimensional schematic representation of the eight seed brain regions selected for analysis. (R) Correlation matrix depicting mean correlation coefficients (*r*‐values) for Sham and IP groups (*n* = 5). ns, not significant; ^*/#^ *p* < 0.05; ^**/##^ *p* < 0.01; ^***/###/△△△^ *p* < 0.001. ^*^compared with Sham group; ^#^compared with IH group; ^△^compared with PgO group.

We first isolated and purified PgO from the culture supernatant of a standard *Pg* strain (ATCC33277). The morphology and particle size of the obtained PgO were characterized by transmission electron microscopy (TEM) and nano flow cytometry (NTA), revealing spherical vesicles with a uniform size and a mean diameter of 67.7 nm (Figure 2A,B; Figure S1A). SDS‐PAGE analysis further confirmed the presence of bacterial proteins within the obtained PgO (Figure S1B).

To investigate the potential comorbidity between periodontitis and cognitive impairment, we evaluated the effects of PgO in combination with an established IH model (Figure 2C). After the 4 week modeling period, IH alone induced significant alveolar bone resorption and attachment loss. Notably, co‐exposure to PgO and IH (IP group) further exacerbated periodontal destruction, resulting in the most severe periodontal phenotype after 4 weeks among all groups. (Figure 2D‐G).

Behavioral assessments were then performed to evaluate locomotor activity, anxiety‐like behavior, and cognitive function. The open‐field test revealed no significant differences in locomotion or anxiety‐related parameters among groups (Figure 2H,I; Figure S2A–F), indicating that subsequent cognitive changes were not confounded by altered activity or anxiety. In the Y‐maze test, neither IH nor PgO alone elicited any significant cognitive impairment, whereas mice subjected to combined IH and PgO exposure (IP group) exhibited marked cognitive decline (Figure 2J,K).

To further delineate the temporal relationship between IH and PgO exposure, we conducted sequential intervention experiments. The introduction of PgO after 4 weeks of pre‐existing IH (IH‐IP group) led to significant cognitive impairment within 2 weeks, whereas the reverse sequence (PgO‐IP group) failed to produce comparable deficits (Figure 2L), highlighting the critical role of pre‐existing IH in sensitizing the brain to PgO‐mediated injury. Although cognitive deficits eventually emerged in all groups by week 8, the IP group consistently displayed the most severe impairment (Figure S2G–J). These findings indicate that periodontitis‐associated PgO accelerates the onset and progression of IH‐induced cognitive dysfunction.

To identify the brain regions particularly vulnerable to IH, we performed whole‐brain c‐Fos staining on optically cleared brain tissues followed by 3 days of IH exposure. The results indicated that neuronal activation was most prominent in the hippocampus, especially within the CA1 subregion (Figure 2M–O; Figure S3 and Videos S1 and S2), consistent with the established sensitivity of CA1 neurons to hypoxic stress [33, 34].

Given the early cognitive decline observed in the IP group at 4 weeks post‐modeling, we employed functional magnetic resonance imaging (fMRI) to assess functional connectivity in the mouse brain. Voxel‐wise functional connectivity analysis using the hippocampal CA1 subregion as the seed point revealed markedly reduced functional connectivity across the brain in the IP group compared with the Sham group (Figure 2P). Furthermore, we selected eight brain regions previously reported to be associated with CA1 activity [35, 36]. A matrix depicting the group‐averaged correlation coefficients (*r*‐values) showed prominent correlations in seed point activity within the Sham group, which were attenuated in the IP group (Figure 2Q,R).

Collectively, these findings suggest that the hippocampal CA1 neurons exhibit exceptional neuronal sensitivity to acute IH, and the chronic IH combined with periodontitis disrupts CA1‐centered functional connectivity, providing a neural basis for the accelerated cognitive impairment observed under conditions of hypoxia‐infection comorbidity.

### 2.2 PgO Exacerbated Oxidative Stress and Inflammation in IH, Accelerating Neuronal and Mitochondrial Damage

**FIGURE 3:**
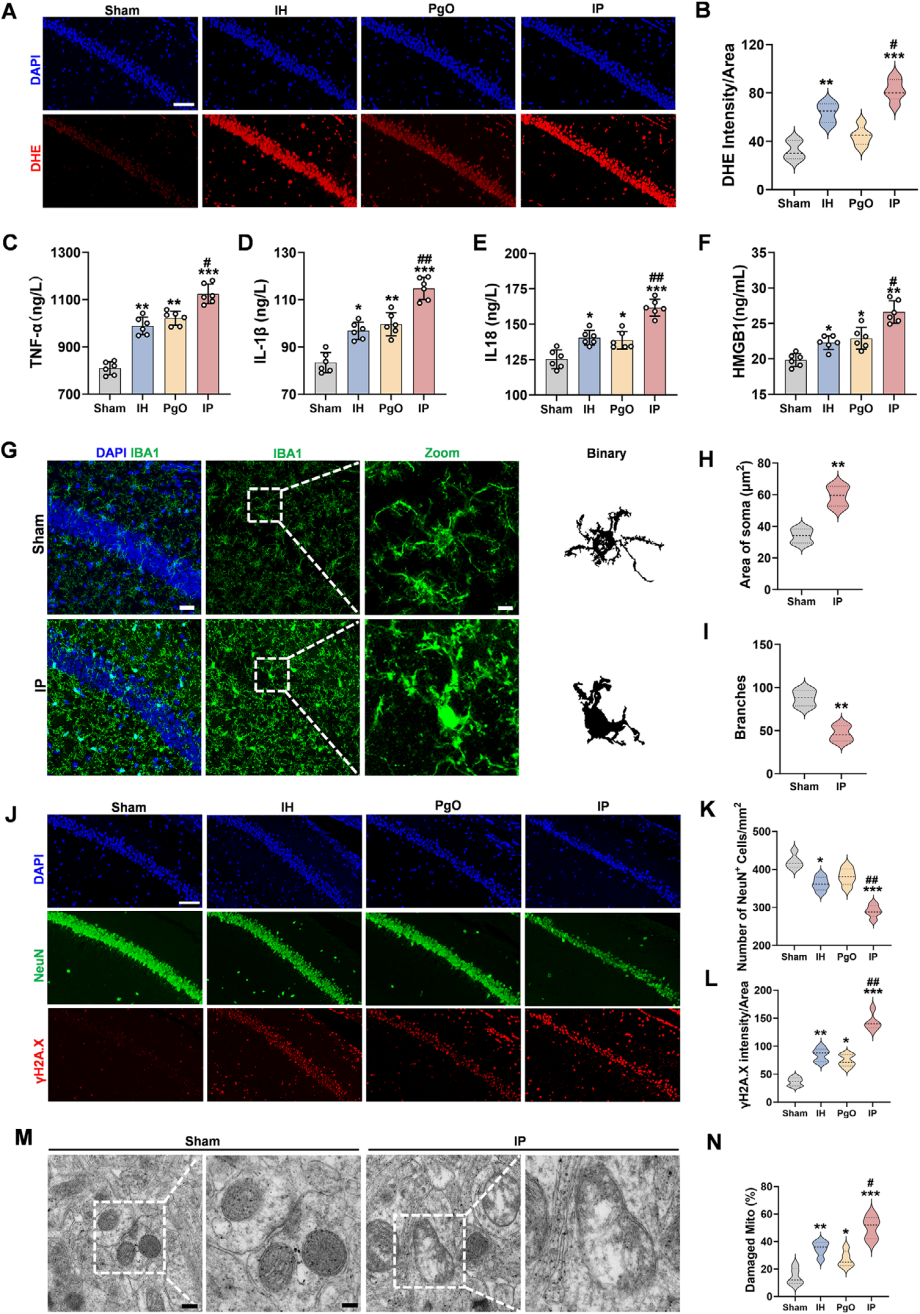
PgO exacerbates hippocampal oxidative stress and neuroinflammation in the context of IH, driving accelerated mitochondrial damage and neuronal loss in the CA1 region. (A, B) Representative images of DHE immunofluorescence staining in the mouse hippocampal CA1 region after 4 weeks of modeling (*n* = 5; scale bar: 50 µm). (C–F) Quantification of hippocampal inflammatory cytokines by ELISA in mice (*n* = 6). (G–I) Representative images of IBA1 immunofluorescence in mouse CA1 hippocampus, accompanied by quantification of microglial morphology, including soma area and branch number (*n* = 5; scale bar: 20, 5 µm correspondingly). (J–K) Immunofluorescence staining of NeuN and γH2A.X in the mouse hippocampal CA1 region (*n* = 5; scale bar: 50 µm). (M, N) TEM images of mitochondria in mouse hippocampal neurons (*n* = 5; scale bar: 250, 100 nm correspondingly). ns, not significant ^*/#/^ *p* < 0.05; ^**/##/^ *p* < 0.01; ^***/###/^ *p* < 0.001. ^*^compared with the Sham group; ^#^compared with IH group.

Subsequently, we investigated the brain damage induced by the comorbidity of IH and periodontitis at the histological level. At the 4‐week endpoint, we assessed oxidative stress and inflammatory responses. Dihydroethidium (DHE) staining revealed a significant increase in reactive oxygen species (ROS) in the hippocampal CA1 region of the IP group compared to the IH group (Figure 3A,B). Furthermore, ELISA measurements of hippocampal and serum samples showed that either IH or PgO alone exposure increased levels of TNF‐α, IL‐1β, IL‐18, and HMGB1, while the co‐treatment (IP) led to the most pronounced upregulation of these inflammatory factors (Figure 3C–F; Figure S4A–D). These data suggest that PgO enhances IH‐induced oxidative stress and inflammatory responses.

We therefore examined microglia, the resident immune cells of the CNS. IBA1 immunostaining showed that the IP group exhibited a higher density of microglia in the CA1 region, with enlarged somata, shortened processes, and a transition to an amoeboid morphology—features indicative of activation (Figure 3G–I; Figure S4E–G). Concurrently, reactive astrogliosis was observed, further supporting a state of hippocampal inflammation and stress (Figure S4H–J).

In addition, neuronal integrity was severely compromised in the IP group. NeuN staining showed a progressive loss of CA1 neurons, ranging from mild in IH‐only mice to extensive in IP‐treated animals (Figure 3J,K). This neuronal degeneration was mechanistically linked to genomic instability, as evidenced by a pronounced increase in γH2A.X^+^ neuronal nuclei, indicating the accumulation of DNA double‐strand breaks (Figure 3L; Figure S4K–M). Ultrastructural analysis by TEM provided the direct pathological link, revealing the most severe mitochondrial abnormalities—including cristae dissolution and vacuolar degeneration—in the hippocampus of the IP group (Figure 3 M,N and Figure S4N).

Taken together, these data establish that PgO dramatically potentiates IH‐induced oxidative stress and neuroinflammatory signaling, culminating in accelerated mitochondrial failure and neuronal degeneration.

### 2.3 PgO Potentiates IH‐Induced Hippocampal Damage via the HIF‐1α/HMGB1/NLRP3 Pathway

**FIGURE 4:**
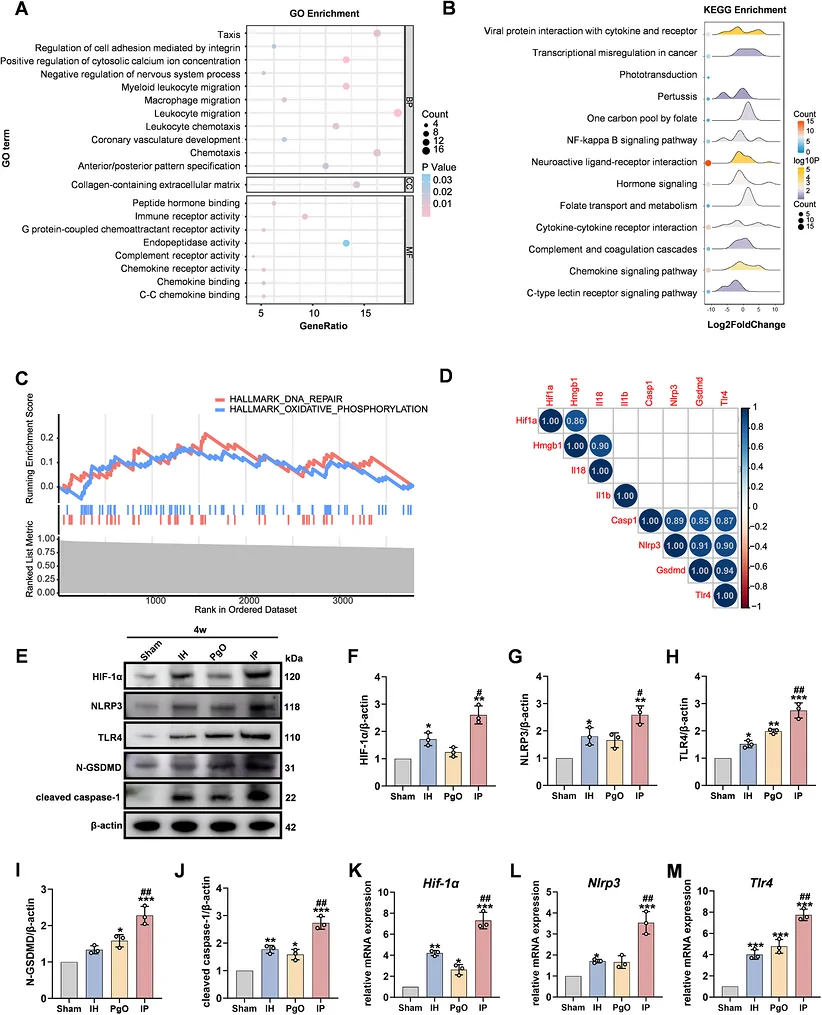
PgO aggravates IH‐induced hippocampal damage by activating the HIF‐1α/HMGB1/NLRP3 axis, which integrates oxidative stress with inflammasome‐driven pyroptosis to accelerate neuronal injury. (A) GO functional enrichment analysis of the hippocampal tissues in the Sham and IP group mice. (B) KEGG pathway enrichment analysis of the hippocampal tissues in Sham and IP group mice. (C) Single‐gene enrichment analysis of NLRP3. (D) Correlation heatmap of HIF‐1α, NLRP3, HMGB1, TLR4 and pyroptosis‐related genes. (E–J) Western blot and (K–M) RT‐qPCR analysis of mouse hippocampal tissues at 4 weeks post‐modeling (*n* = 3). ns, not significant ^*/#^ *p* < 0.05; ^**/##^ *p* < 0.01; ^***/###^ *p* < 0.001. ^*^compared with the Sham group; # compared with IH group.

To further elucidate how PgO exacerbates IH‐induced cognitive impairment, we performed bulk RNA sequencing on mouse hippocampal tissues. Gene ontology (GO) enrichment analysis of differentially expressed genes revealed significant involvement in immune activation processes, including leukocyte chemotaxis and macrophage migration, alongside disrupted neural homeostasis (Figure 4A). KEGG pathway analysis confirmed these findings, showing significant enrichment in chemokine/cytokine signaling and neuroactive ligand–receptor interaction pathways (Figure 4B), which collectively support enhanced immune cell migration and a sustained neuroinflammatory response. Furthermore, the enrichment of the transcriptional misregulation pathway suggests a mechanism for persistent inflammation, potentially involving dysregulated transcription factors such as HIF‐1α. Consistent with these findings, gene expression boxplots confirmed pronounced upregulation of both *Hif1α* and *Nlrp3* (Figure S5A,B).

Notably, our gene enrichment analysis revealed that NLRP3 is specifically associated with DNA damage repair and oxidative phosphorylation pathways rather than general inflammatory pathways (Figure 4C), and the correlation analysis confirmed close associations among HIF‐1α/HMGB1/NLRP3 pathway components, TLR4, and pyroptosis‐related genes (e.g., GSDMD, caspase‐1) (Figure 4D). These findings thus suggest two complementary activation mechanisms: ROS‐induced DNA damage provides persistent activation signals, while combined IH and PgO exposure induces mitochondrial dysfunction, thereby amplifying ROS production and DAMP release (including HMGB1), ultimately leading to NLRP3 inflammasome activation, cleavage of GSDMD and caspase‐1, and pyroptosis.

Consistent with this, hippocampal lysates from the IP group showed increased expression of HIF‐1α, NLRP3, and key pyroptosis markers relative to IH alone at 4 weeks post‐modeling, accompanied by corresponding changes in mRNA levels (Figure 4E–M). By week 8, although overall protein expression was further elevated, the disparity in HIF‐1α expression between the IH and IP groups was no longer significant (Figure S5C–H).

These findings establish the HIF‐1α/HMGB1/NLRP3 axis as a key mechanism through which PgO exacerbates IH‐induced hippocampal neuronal damage, providing a molecular basis for cognitive impairment in IH‐periodontitis comorbidity.

### 2.4 PgO Promotes IH‐mediated Activation of the HIF‐1α/HMGB1/NLRP3 Pathway in HT22 Cells

To dissect the neuron‐intrinsic inflammatory mechanisms independent of systemic factors, we employed the mouse hippocampal neuron cell line HT22 to investigate the impact of IH and PgO co‐exposure. Initial co‐culture experiments established that PgO at 5 µg mL^−1^ did not significantly affect cell viability or proliferation, as confirmed by CCK‐8 assays, and was thus selected for subsequent studies (Figure S6A). Under IH conditions, a significant suppression of HT22 cell proliferation was observed after 3 days, whereas the addition of PgO (5 µg mL^−1^) accelerated this inhibitory effect, manifesting as early as 2 days (Figure S6B–D).

**FIGURE 5:**
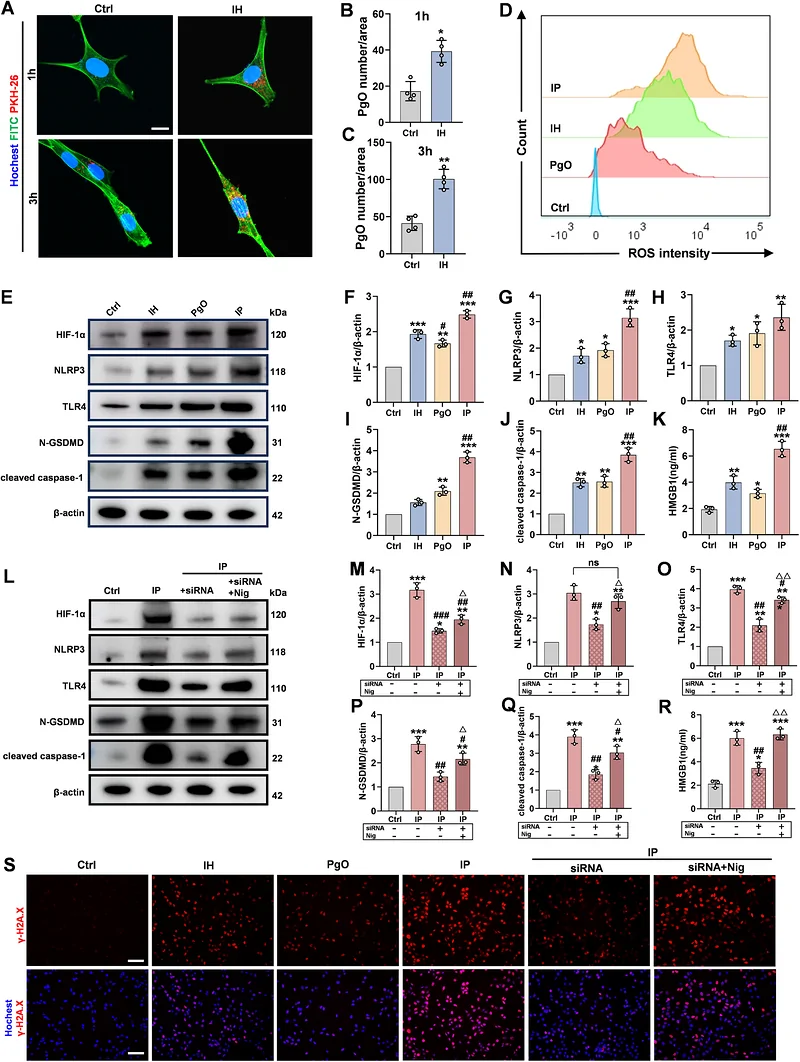
PgO accelerates IH‐induced oxidative stress and inflammatory damage by activating the HIF‐1α/HMGB1/NLRP3 pathway in HT22 cells. (A–C) Confocal microscopy images of HT22 cells after 1 h and 3 h co‐culture with PKH‐26‐labeled PgO (red), showing cellular internalization of PgO (*n* = 4; scale bar: 10 µm). (D) Cellular ROS levels were detected by flow cytometry with DCFH‐DA staining at 12 h post‐treatment. At 48 h post‐treatment, (E–J) protein expression profiling by Western blot using cell lysates. (K) ELISA measurement of HMGB1 concentration in cell culture supernatant. Following HIF‐1α knockdown by siRNA transfection and nigericin stimulation in the IP group cells, (L–Q) protein expression was examined by Western blot using cell lysates. (R) ELISA measurement of HMGB1 concentration in cell culture supernatant. (S) Representative immunofluorescence images of γH2A.X after modeling and treatment of IP group cells with HIF‐1α_siRNA_ transfection and nigericin (*n* = 3; scale bar: 100 µm). ns, not significant ^*/#/△^ *p* < 0.05; ^**/##/△△^ *p* < 0.01; ^***/###/△△△^ *p* < 0.001. ^*^compared with Ctrl group; ^#^compared with IH group (F–K) or with IP group (M–R); ^△^compared with IP+HIF‐1α_siRNA_ group (M–R).

Assessment of PgO uptake using PKH‐26‐labeled vesicles revealed increased internalization into HT22 cells after 1 and 3 h of IH exposure (Figure 5A–C). Following 12 h of IH+PgO co‐treatment, both cellular and mitochondrial ROS levels were significantly elevated (Figure 5D; Figure S6E–H). To determine if PgO exacerbates IH injury via HIF‐1α, we performed Western blot analysis on HT22 cells and observed upregulation of HIF‐1α in the in vitro IP group (IH+PgO co‐exposure) versus IH alone. Additionally, protein levels of NLRP3, TLR4, N‐GSDMD, and cleaved caspase‐1 were increased, supported by corresponding elevated mRNA expression of *Hif1α*, *Nlrp3*, and *Tlr4* and increased HMGB1 secretion (Figure 5E–K; Figure S7A–C). These data indicate that PgO promotes HIF‐1α expression and NLRP3‐mediated inflammatory activation, thereby exacerbating IH‐induced HT22 cell damage. To further interrogate the functional relationship between HIF‐1α and NLRP3 activation, HIF‐1α expression was knocked down via siRNA. Compared to the IP group, the IP+HIF‐1α_siRNA_ group showed significantly reduced HIF‐1α expression, confirming successful knockdown (Figure 5L,M; Figure S7D). Moreover, HIF‐1α silencing markedly reduced the protein levels of NLRP3, TLR4, N‐GSDMD, and cleaved caspase‐1, suppressed extracellular HMGB1 release, and attenuated DNA damage as indicated by γH2A.X staining. In contrast, treatment with the NLRP3 agonist nigericin in the IP group further enhanced the expression of these mediators and exacerbated DNA damage, despite only slightly increasing HIF‐1α levels (Figure 5L–S; Figure S6I). The qPCR results were consistent with the Western blot data (Figure S7E,F).

Collectively, these results demonstrate that PgO augments IH‐induced oxidative stress and promotes inflammatory activation in HT22 cells through the HIF‐1α/HMGB1/NLRP3 pathway, and that HIF‐1α acts upstream of NLRP3 to mediate this pro‐inflammatory cellular injury.

### 2.5 Mitochondria‐Targeted Janus Nanoformulations Attenuate Oxidative Damage in HT22 Cells

**FIGURE 6:**
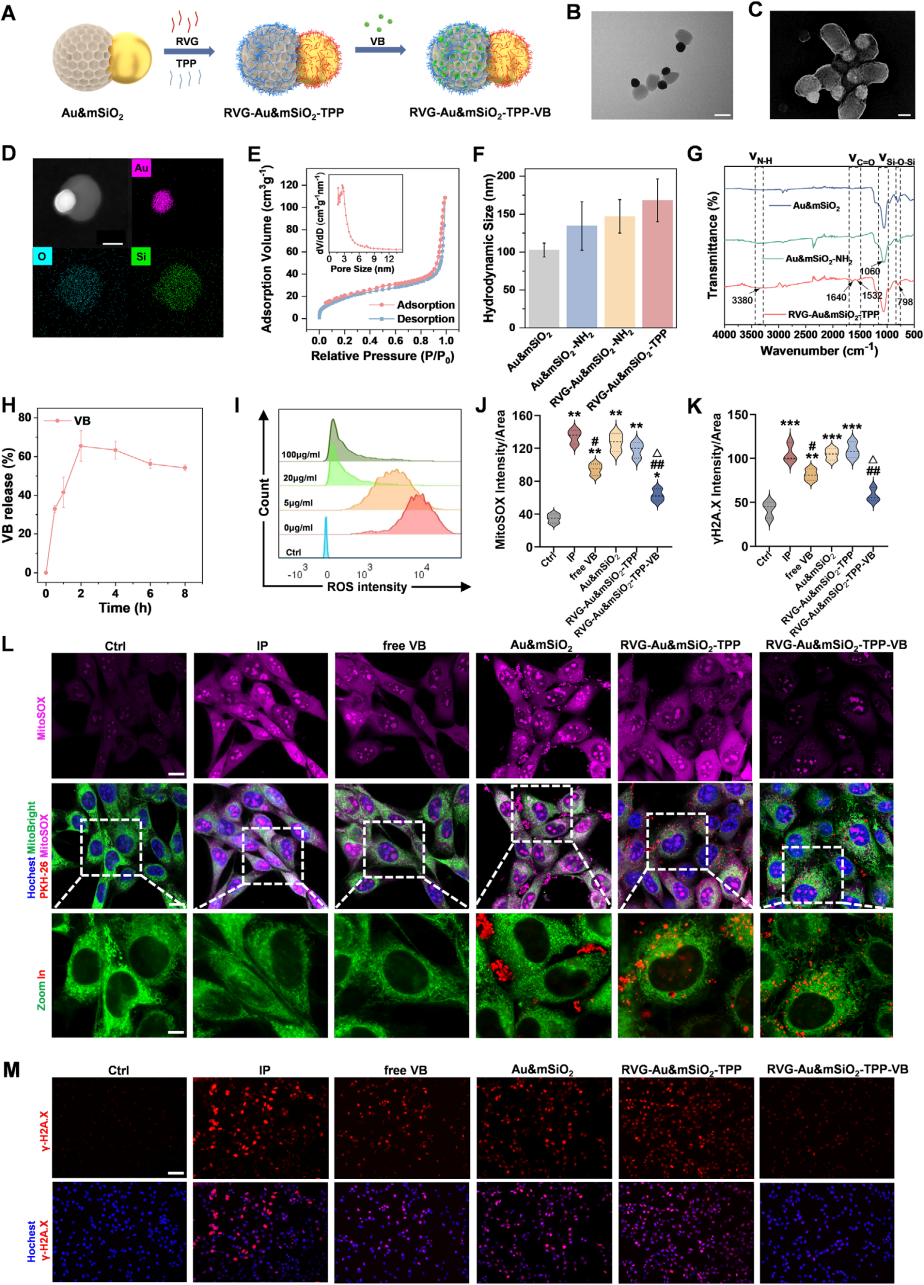
Design, characterization, and mitochondrial antioxidant function of hierarchically targeted Janus nanoformulations. (A) The schematic illustration of the construction of the hierarchical neuron‐to‐mitochondria targeted Janus nanoformulation and VB loading into mesoporous channels. (B, C) TEM and SEM images showing well‐defined Janus nanostructure of the nanoformulations with uniform size and good dispersion (scale bar: 100, 50 nm correspondingly). (D) HAADF‐STEM image and corresponding EDS elemental mappings of the Janus nanoformulations confirming spatial segregation of Au and Si distributions (scale bar: 50 nm). (E) N_2_ adsorption–desorption isotherms and BJH pore size distribution of the Au&mSiO_2_ nanoparticles. (F) Hydrodynamic diameters of Au&mSiO_2_ nanoparticles after stepwise surface functionalization. (G) FTIR spectra confirming successful conjugation of RVG and TPP. (H) In vitro release profile of VB from the Janus RVG‐Au&mSiO_2_‐TPP‐VB nanoformulations (*n* = 3). (I) Flow cytometric analysis of intracellular ROS levels in HT22 cells treated with different concentrations of RVG‐Au&mSiO_2_‐TPP‐VB. (J, K) Quantification of mitochondrial ROS levels (MitoSOX intensity) and DNA damage (γH2A.X intensity) in HT22 cells after different treatments (*n* = 3). (L) Confocal microscopy images showing mitochondrial colocalization of PKH26‐labeled nanoparticles in HT22 cells (scale bar: 10 µm, 5 µm correspondingly). (M) Immunofluorescence staining of γH2A.X demonstrating reduced oxidative DNA damage in HT22 cells treated with Janus RVG‐Au&mSiO_2_‐TPP‐VB nanoformulations compared with other groups (scale bar: 100 µm). Note: ns, not significant ^*/#/△^ *p* < 0.05; ^**/##/△△^ *p* < 0.01; ^***/###/△△△^ *p* < 0.001. ^*^compared with the Ctrl group; ^#^compared with the IP group; ^△^compared with the free VB group.

Based on our findings that mitochondrial ROS plays a central role in neuronal injury within a comorbid mouse model, we sought to develop a nanoplatform capable of selectively scavenging mitochondrial oxidative stress in neurons. To this end, we engineered a hierarchically targeted Janus nanoformulation, denoted as RVG‐Au&mSiO_2_‐TPP‐VB. The Janus Au&mSiO_2_ nanostructure was prepared based on the site‐selective deposition of mSiO_2_ onto one side of an Au nanoparticle through the anisotropic island nucleation and growth strategy [37, 38, 39]. To avoid steric hindrance and spatial competition between different targeting ligands arising from isotropic surface functionalization and achieve hierarchical neuron‐to‐mitochondria targeting, the Au compartment of the Janus nanoparticle was selectively functionalized with the neuron‐targeting RVG peptides, while the mSiO_2_ compartment was conjugated with the mitochondria‐targeting ligand TPP. Motivated by prior reports demonstrating the antioxidant and neuroprotective effects of VB in HT22 cells, VB was loaded into the mesoporous channels, enabling targeted delivery of VB to neuronal mitochondria (Figure 6A).

Morphological characterization via scanning electron microscopy (SEM) and TEM images revealed well‐defined Janus nanostructures with uniform size and good dispersibility (Figure 6B,C). High‐angle annular dark‐field scanning transmission electron microscopy (HAADF‐STEM) and energy‐dispersive x‐ray spectroscopy (EDS) elemental mapping further confirmed the spatially segregated distribution of Au and Si across opposite hemispheres of the Au&mSiO_2_ nanostructure (Figure 6D; Figure S8A), with a quantitative Si/Au atomic ratio of approximately 5:3. Nitrogen adsorption‐desorption measurements showed a Brunauer–Emmett–Teller (BET) surface area of ∼71.9 m^2^ g^−1^ and Barrett–Joyner–Halenda (BJH) pore size of ∼2 nm, indicative of well‐developed mesoporous channels within the mSiO_2_ compartment (Figure 6E).

Successful anisotropic surface modification of the Janus nanoparticles with RVG and TPP was evidenced by characteristic changes in hydrodynamic diameter, ζ‐potential, and Fourier‐transform infrared (FTIR) spectra (Figure 6F,G; Figure S8B). These results are consistent with RVG attachment to the Au compartment via Au─S bonds and TPP conjugation to the mSiO_2_ compartment through amide linkages. Owing to the abundant mesoporous channels, the Au&mSiO_2_ nanocarrier exhibited efficient small‐molecule loading capability. UV–vis absorption analysis indicated a loading capacity of 9.45 µg mg^−1^ for sodium riboflavin phosphate (Figure S8C), and in vitro release studies showed an initial burst release profile, with ∼60% of the payload released within the first 3 h (Figure 6H). After RVG29 modification, an evident spectral change was observed in the low‐wavenumber region (240–280 cm^−^^1^), which is consistent with the appearance of an Au─S‐related vibrational feature (Figure S8D). The circular dichroism (CD) spectra at 0, 2, and 4 days exhibit highly similar profiles, with the main negative band consistently at ∼200 nm and no peak shift or spectral reshaping. These data confirm that RVG29 maintains its conformational stability under the preparation conditions, supporting its subsequent targeting function (Figure S8E).

The biocompatibility of the VB‐loaded hierarchically targeted nanoformulations (RVG‐Au&mSiO_2_‐TPP‐VB) was evaluated using CCK‐8 assays. No significant cytotoxicity was observed in HT22 cells at concentrations up to 100 µg mL^−1^, with cell viability consistently exceeding 90% (Figure S8F). Flow cytometry analysis using DCFH‐DA staining demonstrated that RVG‐Au&mSiO_2_‐TPP‐VB at 20 µg mL^−1^ significantly reduced intracellular ROS levels in HT22 cells (Figure 6I). Therefore, the concentration of 20 µg mL^−1^ was used in subsequent experiments to evaluate the performance of the nanoformulation.

To assess the mitochondrial targeting efficiency, HT22 cells were incubated with PKH‐26‐labeled nanoparticles, including Au&mSiO_2_, RVG‐Au&mSiO_2_, Au&mSiO_2_‐TPP, RVG‐Au&mSiO_2_‐TPP, RVG‐Au&mSiO_2_‐TPP‐VB and Au@mSiO_2_ nanoparticles with a core‐shell structure (Figure S8I). Pronounced mitochondrial colocalization was observed exclusively in Janus structure groups containing TPP, confirming the essential role of Janus structure and TPP in mediating mitochondrial targeting. Functional assessment using MitoSOX staining demonstrated that RVG‐Au&mSiO_2_‐TPP‐VB nanoformulations markedly suppressed mitochondrial ROS levels, outperforming both the non‐targeted nanoparticles and free VB (Figure 6J,L; Figure S8G, H, J).

Consistently, after 48 h of co‐culture, γH2A.X staining demonstrated that RVG‐Au&mSiO_2_‐TPP‐VB nanoformulations conferred superior protection against oxidative DNA damage in HT22 cells compared with all other groups (Figure 6K,M).

### 2.6 Neuron‐Targeted Janus Nanoformulations Ameliorate Cognitive Dysfunction in IP Model Mice

**FIGURE 7:**
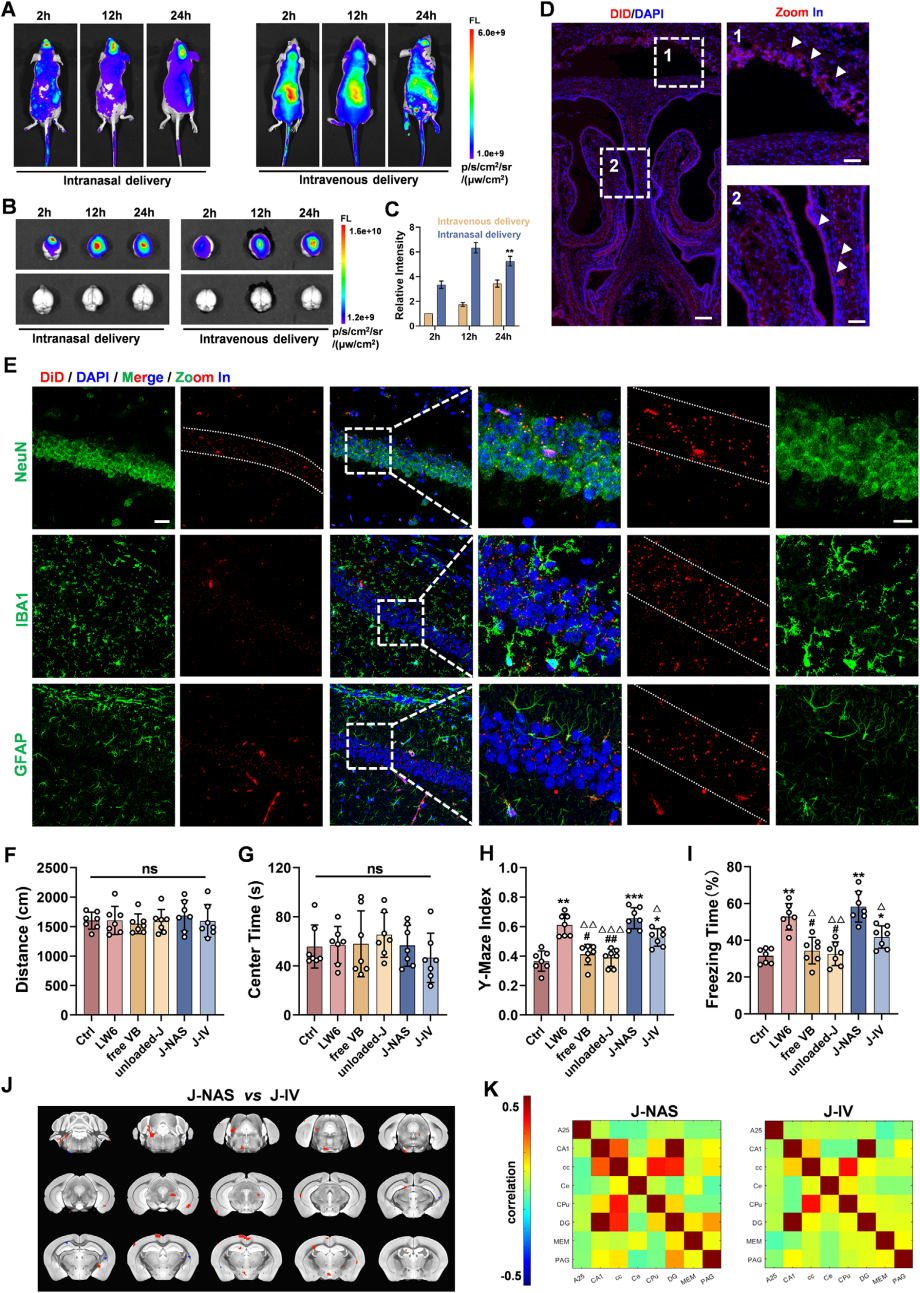
Intranasal delivery of Janus nanoformulations enhances brain accumulation and ameliorates cognitive dysfunction in IP model mice. (A) In vivo distribution and (B, C) brain fluorescence imaging of DID‐labeled Janus nanoformulations at different time points after intranasal/intravenous delivery (*n* = 3). (D) Representative fluorescence image with different magnifications of nasal tissue sections after intranasal administration of DID‐labeled Janus nanoformulations (red). (E) Confocal microscopy images showing colocalization specificity with hippocampal CA1 neurons (NeuN), microglia (IBA1), and astrocytes (GFAP) at 24 h after intranasal delivery of DID‐labeled Janus nanoformulations (*n* = 3; scale bar: 100 µm, 50 µm, 20 µm, 10 µm, correspondingly). Behavioral tests after intervention: (F) open‐field total movement distance, (G) open field center duration, (H) Y‐maze alternation index, (I) Fear conditioning contextual freezing time (*n* = 7). (J) Seed‐based functional connectivity analysis of CA1 using resting‐state fMRI data. Voxels with significant between‐group differences (J‐NAS vs. J‐IV) are projected onto standard brain templates. Red: stronger connectivity in the J‐NAS group relative to the J‐IV group. Blue: weaker connectivity in the J‐NAS group relative to the J‐IV group. (K) Heatmaps of CA1‐centered functional connectivity matrices in J‐NAS and J‐IV groups (*n* = 5). ns, not significant ^*/#/△^ *p* < 0.05; ^**/##/△△^ *p* < 0.01; ^***/###/△△△^ *p* < 0.001. ^*^compared with intravenous delivery group (E) or Ctrl group (H–K); ^#^compared with LW6 group; ^△^compared with J‐NAS group.

In addition to conventional systemic intravenous administration, intranasal delivery was employed to evaluate the brain‐targeting efficiency of RVG‐Au&mSiO_2_‐TPP‐VB Janus nanoformulations. Following intranasal administration, DID‐labeled Janus nanoformulations rapidly entered the brain within 2 h, and exhibited significantly higher levels of brain accumulation at 2, 12, and 24 h compared to intravenous delivery (Figure 7A–C). In contrast, intravenous injection led to substantial hepatic accumulation, whereas intranasal administration markedly reduced liver deposition, with only slightly increased lung accumulation, possibly due to minor inhalation during dosing (Figure S9). Notably, strong fluorescence signals of Janus nanoformulations persisted in the nasal mucosa basal layer for up to 24 h post‐administration, indicating prolonged nasal retention and sustained brain delivery (Figure 7D). Whole‐brain fluorescence imaging further revealed extensive distribution of the Janus nanoformulations throughout the brain (Figure S10A). Immunofluorescence shown in Figure 7E further confirmed predominant colocalization of Janus nanoformulations with hippocampal CA1 neurons (NeuN), with minimal colocalization with microglia (IBA1) and astrocytes (GFAP), demonstrating high selectivity of the Janus nanoformulations in vivo (Figure S11).

The therapeutic efficacy of the Janus nanoformulations was subsequently evaluated in an IP model of mice. Animals received daily intranasal or intravenous administration of the Janus nanoformulations for 4 weeks (denoted as J‐NAS or J‐IV, respectively). Other groups included intranasal free VB group (free VB), unloaded RVG‐Au&mSiO_2_‐TPP Janus nanoparticles group (unloaded‐J), saline group (Ctrl), and treated intravenously with the HIF‐1α inhibitor LW6 group (LW6). Here, the LW6 group served to validate the central role of HIF‐1α in IP‐associated cognitive impairment and to provide a mechanistically defined efficacy benchmark for subsequent nanotherapeutic interventions. After 4 weeks of treatment, behavioral tests showed no significant differences in locomotor ability or anxiety‐like behavior among groups (Figure 7F,G). However, both Y‐maze and fear conditioning tests revealed that Janus formulation treatment significantly improved cognitive performance, with intranasal delivery showing superior or comparable efficacy relative to that of intravenous administration (Figure 7H,I; Figure S12A).

Consistently, the fMRI analysis demonstrated significantly enhanced functional connectivity between the hippocampal CA1 seed point and global brain voxels in both J‐NAS and J‐IV groups compared to the IP group. Notably, the intranasal delivery group exhibited more extensive voxel‐wise connectivity and stronger CA1‐to‐global brain integration, highlighting the advantage of the nose‐to‐brain delivery route (Figure 7J,K; Figure S12B). Histopathological assessment confirmed the biosafety of the nanoformulations, with hematoxylin and eosin (H&E) staining showing intact nasal septal and turbinate mucosa following intranasal administration, as well as no observable microscopic pathological abnormalities in major organs across all treatment groups (Figure S10B,C).

### 2.7 Amelioration of Oxidative Stress, Mitochondrial Dysfunction, and Pyroptosis in the Hippocampal CA1 Region by Janus Nanoformulations

**FIGURE 8:**
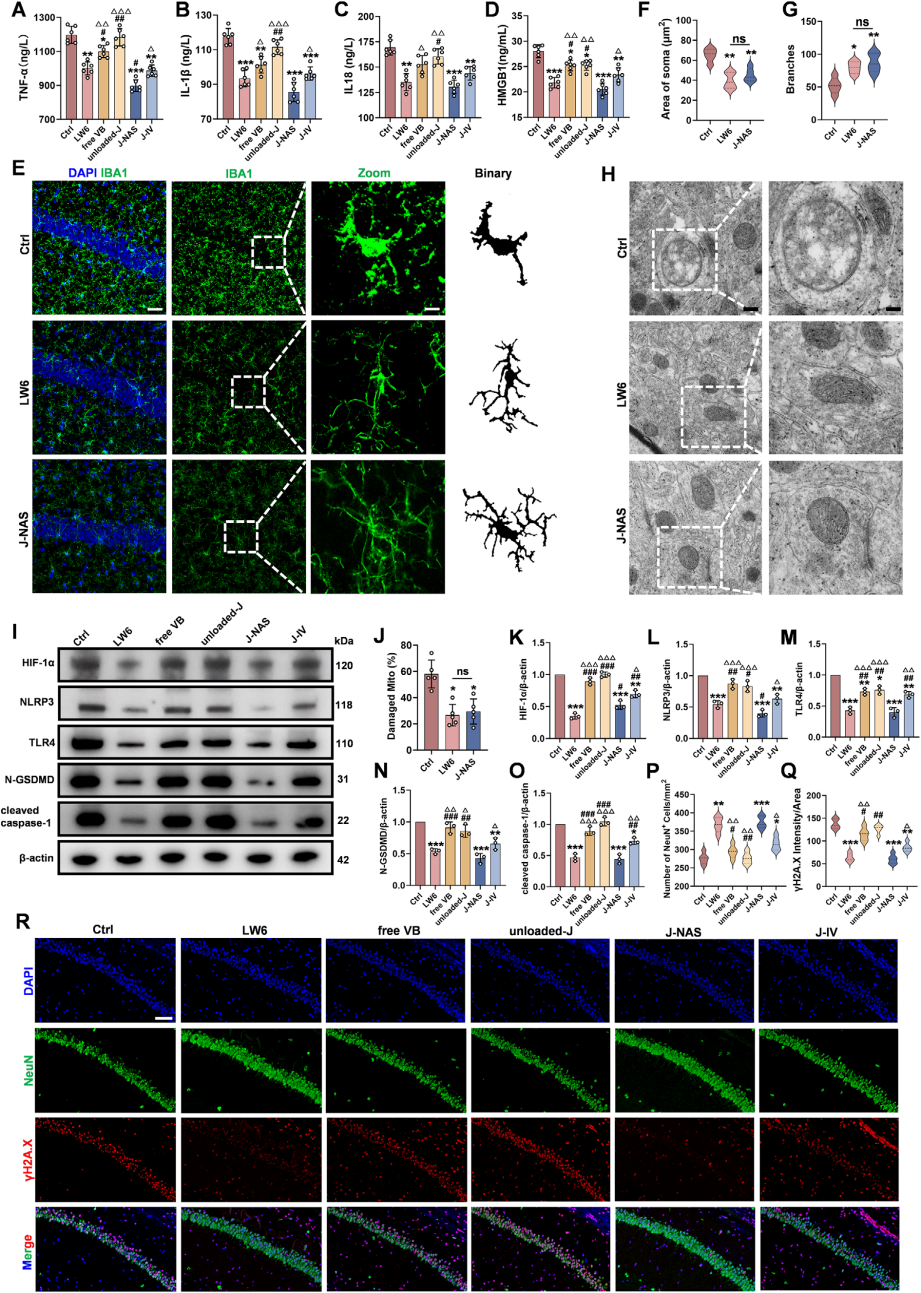
Neuron‐to‐mitochondria targeted Janus nanoformulations attenuate oxidative stress, mitochondrial dysfunction, and pyroptosis in the hippocampal CA1 region. (A–D) ELISA quantification of TNF‐α, IL‐1β, IL‐18, and HMGB1 levels in hippocampal tissue following different treatments (*n* = 6); (E) Representative immunofluorescence images of IBA1‐positive microglia following different treatments in the CA1 region and corresponding binary skeleton analysis (scale bar: 20, 5 µm correspondingly); (F,G) Quantification of microglial morphology, including soma area and branch number (*n* = 5); (H) TEM images showing mitochondrial structure in CA1 neurons (scale bar: 250, 100 nm correspondingly); (I) Representative Western blot images of HIF‐1α, TLR4, NLRP3, N‐GSDMD, and cleaved caspase‐1 expression in hippocampal tissue; (J–O) Quantitative analysis of damaged mitochondria (*n* = 5), and relative protein expression levels (*n* = 3); (P, Q) Quantification of NeuN‐positive neurons in the CA1 subregion and γH2A.X fluorescence intensity (*n* = 5); (R) Representative immunofluorescence images of NeuN and γH2A.X staining in the hippocampal CA1 region across treatment groups (scale bar: 50 µm). Note: ns, not significant; ^*/#/△^ *p* < 0.05; ^**/##/△△^ *p* < 0.01; ^***/###/△△△^ *p* < 0.001. ^*^compared with Ctrl group; ^#^compared with LW6 group; ^△^compared with J‐NAS group.

Further DHE immunofluorescence staining and ELISA analyses demonstrated that treatment with LW6, intranasally administered Janus nanoformulations (J‐NAS), and intravenously administered Janus nanoformulations (J‐IV) all significantly reduced oxidative stress in the hippocampal CA1 region and reduced levels of inflammatory factors, including TNF‐α, IL‐1β, IL‐18, and HMGB1, in both hippocampus tissue and serum. Notably, intranasal delivery of the Janus nanoformulations exhibited superior anti‐inflammatory and antioxidant efficacy compared with intravenous administration (Figure 8A–D; Figure S13A–F). Immunofluorescence staining of IBA1 revealed that both LW6 and J‐NAS suppressed microglial activation and reactive astrogliosis in the hippocampal CA1 area, indicating amelioration of the local neuroinflammatory microenvironment (Figure 8E–G and Figure S13G,H,J,K). Consistent with these findings, TEM imaging showed substantial restoration of mitochondrial ultrastructure following LW6 and J‐NAS treatment, as evidenced by increased mitochondrial number and well‐preserved cristae morphology (Figure 8H,J). In addition, after cessation of intranasal administration, brain Au content decreased from 70.73 mg/kg (0 days post treatment) to 0.31 mg/kg (14 days post treatment), which was no longer significantly different from untreated controls (Figure S13I). These data indicate that Au is largely cleared from the brain within 2 weeks after cessation of treatment.

Western blot analysis further revealed that pharmacological inhibition of HIF‐1α by LW6 robustly downregulated the expression of TLR4, NLRP3, N‐GSDMD, and cleaved caspase‐1 compared to the control group, supporting the role of NLRP3 inflammasome activation as a downstream effector of HIF‐1α signaling in PgO‐aggravated, IH‐induced cognitive impairment (Figure 8I,K–O). Although J‐IV also partially reduced hippocampal expression of HIF‐1α and its downstream pyroptosis‐related proteins, its overall inhibitory effect was less pronounced than that observed in the J‐NAS group. These regulatory trends were further corroborated at the mRNA level by qPCR (Figure S14). Finally, NeuN and γH2A.X immunofluorescence staining indicated that both LW6 and J‐NAS effectively attenuated neuronal DNA damage in the hippocampal CA1 subregion, with significantly greater neuroprotective effects than J‐IV. In contrast, intranasally administered free VB and unloaded Janus nanoparticles showed limited neuroprotection (Figure 8P–R).

## 3 Discussion

Accumulating epidemiological and clinical evidence has established IH and periodontitis as significant, independent risk factors for cognitive decline and neurodegenerative disorders (e.g., AD) [40, 41, 42, 43]. Notably, these conditions exhibit a high degree of clinical co‐occurrence, suggesting that their combination may accelerate neurodegeneration to a greater extent than either condition alone. However, previous investigations have largely confined themselves to studying these etiologies in isolation. Our study bridges this critical knowledge gap by providing the first direct experimental evidence that outer membrane vesicles from the keystone periodontal pathogen *Porphyromonas gingivalis* (PgO) exacerbate IH‐induced hippocampal damage and cognitive impairment. We delineate a coherent molecular cascade, the HIF‐1α/HMGB1/NLRP3 axis, through which PgO amplifies IH‐induced oxidative stress, culminating in inflammatory pyroptosis of vulnerable CA1 neurons. This work establishes a novel etiological paradigm for comorbidity‐associated cognitive dysfunction, moving beyond correlative observations to a defined mechanism.

Currently, effective interventions for cognitive impairment resulting from this comorbidity remain limited. Continuous positive airway pressure therapy serves as the standard treatment for IH [44]; however, its impact on patients’ quality of life and its efficacy in mitigating associated neuroinflammation and cognitive decline require further validation. Similarly, conventional periodontal therapy, though effective in controlling local gingival inflammation, demonstrates minimal influence on inflammatory mediators that have already reached the central nervous system. Therapeutic translation for such complex, multi‐factorial neurological conditions faces a formidable barrier: the lack of delivery systems capable of precise, hierarchical targeting from the organ to the subcellular level. Conventional nanoparticle modifications often result in random, crowded ligand presentation, leading to steric hindrance and compromised targeting efficacy [45, 46, 47]. As shown in Figure S8J, the Au@mSiO_2_‐RVG‐TPP‐VB group showed very limited colocalization with mitochondria (MitoBright), indicating that the mitochondrial targeting function of TPP is hindered by the random distribution of RVG in the co‐functionalized system, thereby failing to efficiently direct the particles to mitochondria. Moreover, the Au@mSiO_2_‐RVG‐TPP‐VB group exhibited limited capacity to scavenge mitochondrial ROS (MitoSOX), further confirming that mutual interference between the two targeting moieties in the mixed functionalization strategy compromises the overall therapeutic efficacy. To overcome this, we engineered an innovative, spatially programmed Janus nanoarchitecture (RVG‐Au&mSiO_2_‐TPP). The key design innovation lies in the asymmetric segregation of targeting motifs: the neuron‐specific RVG peptide is exclusively conjugated to the Au compartment, while the mitochondria‐homing TPP moiety is anchored on the opposing mSiO_2_ compartment. This strategic spatial decoupling eliminates mutual interference, preserves ligand functionality, and enables a sequential “neuron‐first, mitochondria‐next” targeting logic, a significant advancement over conventional isotropic nanocarriers [48, 49].

Furthermore, our approach exemplifies a mechanism‐informed strategy for therapeutic cargo selection and delivery. Bioinformatic analysis of damaged hippocampal tissues revealed significant enrichment in the “folate transport and metabolism” pathway (Figure 4B), a process critically dependent on riboflavin (vitamin B2, VB) as a cofactor for methylenetetrahydrofolate reductase (MTHFR) [50, 51]. Deficiency in VB impairs MTHFR function, leading to metabolic disturbances characterized by reduced generation of active folate (5‐MTHF), elevated homocysteine levels, and compromised methylation capacity—processes critically involved in neuronal maintenance and DNA repair [52]. Concurrently, clinical observations indicate reduced VB levels in AD patients, while VB supplementation has demonstrated benefits in preventing and treating cognitive impairment [53]. Therefore, we rationally selected VB not merely as a generic antioxidant but as a metabolic corrector to address the pathway dysfunction identified in our model. Encapsulating VB within the mesoporous silica compartment ensures its protected delivery precisely to the site of paramount pathology, i.e., neuronal mitochondria. The dramatic therapeutic efficacy of our nanoformulations, achieved with a VB dose approximately one‐tenth of that required for free drug administration, underscores the power of this targeted, mechanism‐driven delivery strategy. It demonstrates that therapeutic outcomes are governed not just by drug potency, but dominantly by spatiotemporal delivery precision [54, 55].

It should be noted that the present study primarily focuses on neuron‐intrinsic molecular mechanisms, revealing the central role of the HIF‐1α/HMGB1/NLRP3 axis in neuronal injury. However, the central nervous system is a complex network composed of neurons, microglia, astrocytes, vascular endothelial cells, and other cell types. Microglia, as the resident immune cells of the central nervous system, are key initiators and amplifiers of neuroinflammatory responses. Astrocytes play important roles in maintaining blood‐brain barrier integrity, regulating glutamate homeostasis, and secreting neurotrophic factors. Under the synergistic insult of IH and PgO, whether glial cells are activated via the same HIF‐1α/HMGB1/NLRP3 pathway, and how activated microglia/astrocytes engage in inflammatory signaling crosstalk with damaged neurons leading to neuronal injury, represent important directions that have not been deeply explored in the current study but warrant further investigation in the future.

Despite these promising findings, several limitations warrant attention in future research. First, while preliminary biosafety assessments of nasal mucosa and major organs showed no significant abnormalities, more systematic toxicological studies are required to evaluate potential local immune responses or neurotoxicity. Long‐term clearance kinetics (>2 weeks) and long‐term neurotoxicity (up to 2∼3 months after cessation of treatment) of the gold nanoparticles have not been systematically evaluated in the present study. This represents a limitation of our work and will be necessary to address prior to any future clinical translation. Furthermore, clinically, patients with IH often present with concomitant hypercapnia, which can independently affect cognitive function. The open‐circuit gas exchange system used in this study effectively eliminated CO_2_ retention, thereby enabling a more precise delineation of the pathophysiological role of IH per se in cognitive impairment. For patients with IH complicated by hypercapnia, the underlying pathological mechanisms may be more complex, and future studies should further explore the synergistic effects of hypoxia and hypercapnia. Finally, whether this nanoplatform can serve as a versatile system for delivering other neuroprotective agents (e.g., antioxidants or anti‐inflammatory drugs) to address various neurological disorders represents a compelling direction for further investigation.

## 4 Conclusion

In summary, this study reveals a previously underappreciated mechanistic link between intermittent hypoxia and periodontitis in driving cognitive impairment. Using a comorbid mouse model, we demonstrate that PgO exacerbates IH‐induced hippocampal oxidative stress, neuroinflammation, and mitochondrial dysfunction, ultimately leading to neuronal damage and cognitive decline. Mechanistically, this pathological process is mediated through amplification of the HIF‐1α/HMGB1/NLRP3 signaling axis, which promotes inflammatory pyroptosis in vulnerable hippocampal CA1 neurons. Guided by this mechanism, we developed a Janus RVG‐Au&mSiO_2_‐TPP‐VB nanoformulation enabling hierarchical neuron‐to‐mitochondria targeting, in which VB is encapsulated within mSiO_2_ for protected mitochondrial delivery. Intranasal administration of this nanoformulation achieves efficient brain and neuronal accumulation, robust suppression of mitochondrial oxidative stress and neuroinflammation, preservation of mitochondrial integrity, and significant restoration of hippocampal function and cognition, outperforming intravenous delivery and free VB administration. Collectively, our findings not only elucidate how hypoxia and chronic peripheral infection cooperatively accelerate neurodegeneration but also establish a mechanism‐driven, hierarchically targeted nanotherapeutic strategy for treating comorbidity‐associated cognitive dysfunction. This work provides conceptual and translational insights for addressing complex neurological disorders arising from systemic hypoxia‐inflammation interactions.

## 5 Experimental Section

Detailed procedures for the preparation and characterization of RVG‐Au&mSiO_2_‐TPP‐VB nanoformulations, along with materials, protocols for cell experiments, imaging assays, and animal studies, are provided in the Supporting Information. All animal experiments were approved by the Animal Care and Use Committee of Shanghai Jiao Tong University (Approval No.: A2024507) and performed in accordance with the ARRIVE guidelines and the National Institutes of Health (NIH) standards for the care and use of laboratory animals [56].

## Author Contributions

**Kai‐Run Zhang**: conceptualization, methodology, software, data curation, investigation, validation, formal analysis, visualization, project administration, writing – original draft. **Tianbao Zhu**: conceptualization, methodology, software, data curation, investigation, validation, formal analysis, visualization, project administration, writing – original draft. **An‐Qi Liu**: conceptualization, writing – review and editing, funding acquisition, project administration, resources, investigation, data curation, supervision. **Jia‐Jia Deng**: conceptualization, methodology, investigation, visualization, resources. **Ya‐Li Han**: software, data curation, formal analysis, visualization. **Sheng‐Cai Qi**: resources, supervision, project administration, conceptualization. **Xue Liu**: resources, methodology, visualization, investigation. **Tian‐Yi Jiang**: software, data curation, formal analysis, visualization. **Ming‐Rui Zhai**: investigation, validation, methodology. **Yu‐Ying He**: investigation, validation, methodology. **Xiu‐Juan Li**: visualization, software, methodology. **Hong‐Ting Da**: investigation, validation, formal analysis, writing – review and editing. **Rui Xu**: investigation, validation, formal analysis, writing – review and editing. **Xiaomin Li**: conceptualization, funding acquisition, methodology, writing – review and editing, project administration, supervision, resources. **Jun Gao**: conceptualization, methodology, supervision, resources, project administration, writing – review and editing, funding acquisition. **Yue‐Hua Liu**: conceptualization, methodology, supervision, resources, project administration, writing – review and editing, funding acquisition.

## Funding

The National Key R&D Program of China (2024YFC3406401, 2022YFC2705200), the National Key Clinical Program on Orthodontics (GJLCZDZK‐2023‐01), the Shanghai Top Priority Research Center Program (2023ZZ02009), National Natural Science Foundation of China (22525501, 22475049, 22405051, 32371053, 82571143), Shanghai Pilot Program for Basic Research‐Fudan University (22TQ004), Science and Technology Commission of Shanghai Municipality (2024ZDSYS02, 25PY2600100), Fundamental research program funding of Ninth People's Hospital Affiliated to Shanghai Jiao Tong University School of Medicine (JYZZ248). The Scientific Research Project of Anhui Provincial Department of Education (2025AHGXZK40288).

## Conflicts of Interest

The authors declare no conflicts of interest.

## Supporting information




**Supporting File 1**: adma73920‐sup‐0001‐SuppMat.docx.

**Supporting File 2**: adma73920‐sup‐0002‐VideoS1.mp4.

**Supporting File 3**: adma73920‐sup‐0003‐VideoS2.mp4.

## Data Availability

All data supporting the findings of this study are included in this article and its Supporting Information files. The data supporting the findings of this study are available from the corresponding author on reasonable request.
