# Supplementary material for: Mechanism‐Driven Nanoformulations for Cognitive Impairment Induced by Intermittent Hypoxia and Periodontitis
Source: Adv Mater. 2026 Jul 7;38(49):e73920. doi: 10.1002/adma.73920 (PMC13532445; doi:10.1002/adma.73920)
Supplement: Supplementary file 1 [file ADMA-38-e73920-s002.docx]

Supplementary information for

**Mechanism-Driven Nanoformulations for Cognitive Impairment Induced by Intermittent Hypoxia and Periodontitis**

*Kai-Run Zhang, Tian-Bao Zhu, An-Qi Liu, Jia-Jia Deng, Ya-Li Han, Sheng-Cai Qi, Xue Liu, Tian-Yi Jiang, Ming-Rui Zhai, Yu-Ying He, Xiu-Juan Li, Hong-Ting Da, Rui Xu, Xiao-Min Li*, Jun Gao*, Yue-Hua Liu**

**Figure S1.** (A) NTA and (B) SDS-PAGE analysis of PgO.

**Figure S2.** At 4 weeks post-modeling: OFT (A) trajectory, (B) velocity, (C) periphery time, (D) center time. At 6 weeks post-modeling: (E) OFT velocity. At 8 weeks post-modeling: OFT (F) center time, (G) velocity; novel object recognition (H) trajectory, (I) discrimination index; contextual fear conditioning (J) freezing time, (K) extinction curve (n=7). ns, not significant * P < 0.05; ** *P* < 0.01. *** *P* < 0.001. * compared with Sham group.

**Figure S3.** (A) Before tissue clearing, (B) after tissue clearing, and (C) with multi-well plate. Expression of c-Fos in (D) different brain regions and (E) hippocampal subregions in Sham and IH groups (n=3).

**Figure S4.** At 4 weeks post-modeling: (A-D) serum inflammatory factor levels by ELISA; immunofluorescence images of (E-G) microglia and (H-J) astrocytes in hippocampal region. Immunofluorescence staining of NeuN and γH2A.X in the mouse hippocampal CA1 region at 8 weeks post-modeling (n=5; scale bar: 50 μm). ns, not significant */# *P* < 0.05; **/## *P* < 0.01; ***/### *P* < 0.001. * compared with Sham group; # compared with IH group.

**Figure S5.** (A, B) Box plots of *Hif1α* and *Nlrp3* gene expression; (C-H) western blot analysis of protein expression in mouse hippocampus at 8 weeks post-modeling. ns, not significant */# *P* < 0.05; **/## *P* < 0.01; ***/### *P* < 0.001. * compared with Sham group; # compared with IH group.

**Figure S6.** (A) Cell viability by CCK-8 assay after 24 h co-culture with varying PgO concentrations. (B) Proliferation curve of HT22 cells with 5 μg mL^-1^ PgO. (C) Proliferation curve under IH conditions. (D) Proliferation curve with 5 μg/mL PgO + IH (n=4). (E, G) Mitochondrial ROS (MitoSOX) confocal images and fluorescence intensity analysis. (F, H) Cellular ROS (DCFH-DA) fluorescence images and intensity analysis. (I) Fluorescence intensity analysis of γH2A.X staining (n=3, scale bar: 10 μm, 100 μm correspondingly). ns, not significant */#/△ *P* < 0.05; **/##/△△ *P* < 0.01; ***/###/△△△ *P* < 0.001. * compared with Ctrl group (G-I); # compared with IH group (G, H) or with IP group (I); △ compared with IP+HIF-1α_siRNA_ group.

**Figure S7.** (A-C) mRNA levels of *Hif1α*, *Nlrp3* and *Tlr4* after modeling and (D-F) treatment with HIF-1α_siRNA_ and nigericin (n=3). ns, not significant */#/△ *P* < 0.05; **/##/△△ *P* < 0.01; ***/###/△△△ *P* < 0.001. * compared with Ctrl group; # compared with IH group (A-C) or with IP group (D-F); △ compared with IP+HIF-1α_siRNA_ group.

**Figure S8.** (A) The EDS, (B) ζ-potential and (C) UV-Vis absorption analysis of constructed asymmetric nanoparticles. (D, E) Biocompatibility assessment (n=5). (F, G) The ability of different nanoparticles to alleviate oxidative stress in HT22 cells. (H) The capacity of RVG-Au&mSiO_2_ and TPP-Au&mSiO_2_ to target mitochondria and reduce mitochondrial ROS (n=3; scale bar: 100 μm, 10 μm, 5 μm correspondingly). ns, not significant */#/△ *P* < 0.05; **/##/△△ *P* < 0.01; ***/###/△△△ *P* < 0.001. * compared with Ctrl group (F); # compared with IP group; △ compared with free VB group.

**Figure S9.** Distribution of RVG-Au&mSiO_2_-TPP-VB in heart, liver, spleen, lung and kidney at different time points after (A, B) intranasal or (C, D) intravenous delivery in mice (n=3, scale bar: 500 mm).

**Figure S10.** (A) Distribution of DID-labeled RVG-Au&mSiO_2_-TPP-VB in different brain regions following intranasal delivery. H&E staining images of (B) turbinate, nasal septum and (C) other major organs after intervention treatment (scale bar: 1 mm, 50 μm, 50 μm and 50 μm correspondingly).

**Figure S11.** Representative confocal images showing colocalization of DID-labeled RVG-Au&mSiO_2_-TPP-VB with neurons, microglia, and astrocytes in (A) hippocampal DG region and (B) cerebral cortex after intranasal delivery (scale bar: 20 μm and 10 μm correspondingly).

**Figure S12.** (A) OFT movement trajectories of mice after intervention treatment. (B) Seed-based functional connectivity analysis of CA1 using resting-state fMRI data. Voxels with significant between-group differences (J-NAS/J-IV *vs*. IP) are projected onto a standard brain templates. Red: stronger connectivity in J-NAS/J-IV group relative to IP group. Blue: weaker connectivity in J-NAS/J-IV group relative to IP group.

**Figure S13.** After intervention: (A, F) oxidative stress levels in hippocampal CA1 region (n=5; scale bar: 50 μm); (B-E) serum inflammatory factor levels (n=6); immunofluorescence staining of (G, I) microglia and (H, J) astrocytes in hippocampal region (n=5; scale bar: 200 μm). ns, not significant */#/△ *P* < 0.05; **/##/△△ *P* < 0.01; ***/###/△△△ *P* < 0.001. * compared with Ctrl group; # compared with LW6 group; △ compared with J-NAS group.

**Figure S14.** mRNA levels of *Hif1α*, *Nlrp3* and *Tlr4* after intervention treatment (n=3). ns, not significant */#/△ *P* < 0.05; **/##/△△ *P* < 0.01; ***/###/△△△ *P* < 0.001. * compared with Ctrl group; # compared with LW6 group; △ compared with J-NAS group.

**Experimental Section**

*Isolation and characterization of PgO*

PgO were efficiently isolated using differential centrifugation. Briefly, bacterial culture was first centrifuged at 500 g for 20 min, and the supernatant was collected. The supernatant was then centrifuged at 2500 g for 20 min to remove bacterial debris. After filtration through a 0.22 μm membrane, the supernatant was subjected to ultracentrifugation at 100,000 g for 70 min to obtain the final PgO pellets. For morphological analysis, PgO were fixed with glutaraldehyde. A 5-10 μL aliquot of the PgO suspension was dropped onto a copper grid and adsorbed for 10 min at room temperature. Excess liquid was removed using filter paper, followed by staining with 10 μL of 2% phosphotungstic acid (pH 6.5) for 2 min. The grid was air-dried, and PgO morphology and size were observed by transmission electron microscopy (TEM). For nanoparticle tracking analysis (NTA), the isolated PgO were diluted 100-fold with ultrapure water and analyzed using a ZetaView system. Particle concentration within specific size ranges was determined using the ZetaView software. Protein marker identification was performed by extracting proteins from PgO. Following standard western blot procedures, proteins such as RgpA, Kgp, and HagA were detected using a mouse anti-*Porphyromonas gingivalis* monoclonal antibody (1:100, 61BG1.3, DSHB).

*Cell culture*

The mouse hippocampal neuronal cell line HT22 (Corning Costar, MA, USA) was cultured in DMEM (Gibco, MA, USA) with 10% fetal bovine serum (FBS). The cells were all incubated at 37 °C with 5% CO_2_. All cell lines used in this study have been authenticated and confirmed free of common misidentifications listed in the Cellosaurus database.

*Intermittent hypoxia (IH) exposure*

The protocol was optimized based on our preliminary studies. The experiments were conducted using a professional IH system (Oxycycler Model A84XOV, Biospherix, Redfield, NY), equipped with nitrogen and oxygen generators, oxygen sensors, and circulation fans. Mice were placed daily in the IH chamber from 9:00 to 17:00 for exposure. During modeling, the controller injected nitrogen to reduce the chamber oxygen concentration to 6 ± 1% (maintained for 30 s), followed by oxygen injection to restore the concentration to 21% (maintained for 30 s), constituting one complete hypoxia cycle. Sham group mice were housed under normoxic conditions.

For in vitro IH treatment, a cyclic culture protocol alternating between normoxia (21% O_2_, 20 min) and hypoxia (1% O_2_, 40 min) was applied. Cells in the IH + PgO group were treated with a subtoxic dose of PgO (5 μg mL^-1^) during IH exposure. Control group cells were cultured under normoxic conditions and treated with PBS.

*Animals*

This study utilized 8-week-old male C57BL/6J mice. Mice were housed under controlled environmental conditions with a 12h light/dark cycle and provided with ad libitum access to food and water. Animals were randomly assigned to experimental groups, and all procedures were performed following standardized laboratory protocols including randomization and blinding procedures.

*Mouse models*

The IH mouse model was established as previously described. For the chronic periodontitis (PgO) model, mice received palatal gingival injections of PgO (3×10^8^ particles) adjacent to the maxillary second molars three times per week. The IH+PgO (IP) model group was established by subjecting mice to both IH exposure and concurrent PgO injections. Sham control mice were maintained under normoxic conditions and received gingival injections of an equivalent volume of physiological saline. For interventions, the following treatment groups were established concurrently with IH+PgO exposure:

LW6 group: daily intravenous injection of LW6 (5 mg kg^-1^).

free VB group: intranasal delivery of VB (5 mg kg^-1^).

unloaded-J group: intranasal delivery of unloaded RVG-Au&mSiO_2_-TPP nanoparticles (50 mg kg^-1^).

J-NAS group: intranasal delivery of RVG-Au&mSiO_2_-TPP-VB nanoformulations (50 mg kg^-1^), with an effective VB content of approximately 0.5 mg kg^-1^.

J-IV group: intravenous delivery of RVG-Au&mSiO_2_-TPP-VB nanoformulations (50 mg kg^-1^), with an effective VB content of approximately 0.5 mg kg^-1^.

Control group: intranasal and intravenous delivery of equivalent volumes of physiological saline.

*Behavioral tests*

Open Field Test (OFT): Mice were individually placed in a corner of a 50 cm × 50 cm open-field arena surrounded by opaque walls and allowed to freely explore for 5 min. Their movement was recorded and automatically analyzed using the ANY-Maze video tracking system. Parameters including total distance traveled, time spent in the central zone, and velocity were quantified.

Y-Maze Test: Each mouse was placed at the center of a Y-maze with three identical arms (35 cm × 5 cm × 5 cm) and allowed to explore freely for 5 min. The number of entries into each arm and the sequence of arm visits were recorded. Spontaneous alternation performance was calculated as the percentage of successive entries into three different arms.

Fear Conditioning (FC): On Day 1, after a 3 min acclimation period in the conditioning chamber, mice received one paired auditory cue (28 s) and foot shock (2 s, 0.75 mA). On Day 2, mice were placed back into the unchanged context for 3 min without any stimulation, and freezing behavior was recorded. They were then returned to their home cages for 1 h, followed by a 21 min re-exposure to the context without shock, and again returned to their cages for 21 min. This cycle was repeated three times. On Day 3 and Day 4, mice were again placed in the unchanged context for 3 min, and freezing duration was recorded on each day.

Novel Object Recognition Test (NORT): it was performed in a white-lit environment following a three-phase protocol. In the initial habituation phase, mice were allowed to freely explore the testing chamber (40 cm × 40 cm × 40 cm) for 10 min to acclimate to the experimental setting. During the subsequent training phase, two identical objects were positioned symmetrically in the arena, and each mouse was introduced to the center of the space to freely investigate these objects for 10 min. After a 24 h interval, one familiar object was replaced with a novel object of distinct shape and texture, and the mouse was again placed in the center for free exploration. The duration spent by the mouse within a 15 cm radius of the familiar object was recorded as Tf, while that near the novel object was recorded as Tn. A discrimination index (DI) was then calculated as: (Tn – Tf) / (Tn + Tf) × 100%.

*fMRI*

MRI was conducted on a 9.4T Bruker BioSpec 94/30 USR system, equipped with a 440 mT m^-1^ gradient set, an 86-mm volume transmit RF coil, and a single-channel surface head coil. Under anesthesia maintained by 1.5% isoflurane inhalation, mice were secured in a dedicated holder using tooth and ear bars to ensure stable positioning during data acquisition. Physiological parameters were continuously monitored throughout the procedure. T2-weighted images were obtained with a 2D RARE sequence, configured with the following parameters: repetition time (TR) = 2500 ms, echo time (TE) = 33 ms, field of view (FOV) = 20 × 20 mm, matrix size = 256 × 256, and 22 contiguous slices with a thickness of 0.7 mm.

*Tissue clearing*

Following sample collection, tissues were fixed overnight at 4 °C with gentle agitation in 4% paraformaldehyde (PFA). After fixation, samples were washed 2-3 times with 1× PBS, each wash lasting 2 h. Tissue clearing was performed using the Tissue Clearing Kit (NH-CR-230701, Nuohai Bioscience Instrument Co., Ltd., Shanghai). Fixed samples were immersed in 20 mL of lipid removal solution within centrifuge tubes and subjected to gentle agitation at 4 °C for 7-8 days, with the solution refreshed every 2 days. The delipidated samples were then transferred to 1× PBS and washed at room temperature for 2 h, repeated three times. Finally, samples were immersed in matching solution for refractive index matching until full transparency was achieved, prior to optical 3D imaging. 3D image acquisition was conducted using the Nuohai LS18 light-sheet microscope (Nuohai Bioscience Instrument Co., Ltd., Shanghai; laser channels: 405, 488, 561, 637 nm). For whole-sample 3D fluorescence imaging, each field of view was tiled four times using a 1×/0.25NA air objective (Olympus MVPLAPO). Under the selected imaging conditions, the microscope magnification was set to 4 ×, achieving a spatial resolution of 3.3 × 3.3 × 7 µm^3^. Data were processed using Amira (Thermo Fisher Scientific, USA), followed by registration of mouse brain images to the standard Allen Brain Atlas (CCFv3). Signal segmentation within the mouse brain was performed using the segmentation module in Amira 2021.2. Finally, statistical analysis of signals across different brain regions was carried out.

*Micro-CT*

Alveolar bones were scanned using a SkyScan1272 system (Bruker, Germany). To prevent dehydration during the procedure, all samples were wrapped in non-polyvinylchloride (non-PVC) parafilm. The imaging acquisition was conducted with the following parameters: an image pixel size of 18 μm, a source voltage of 60 kV, a source current of 166 μA, a rotation step of 0.4°, and an exposure time of 1400 ms.

*Hematoxylin-Eosin (H&E) staining*

Maxilla specimens were fixed in 4% formalin (Shanghai Reith Biotechnology, China) and subsequently embedded in paraffin. From the resulting blocks, 4 μm-thick sections were obtained. Prior to staining, these sections underwent deparaffinization in xylene and were then rehydrated through a graded alcohol series. Finally, the sections were stained using hematoxylin and eosin (H&E, Shanghai Reith Biotechnology, China).

*Immunofluorescence staining*

Mouse brains were fixed in 4% PFA for 4-6 h, cryoprotected by immersion in a sucrose gradient, and finally embedded in OCT compound. Using a cryotome (Leica CM1950), 30 µm-thick coronal sections were prepared. These sections, along with cell samples that had been fixed for 15 min, underwent permeabilization and blocking. All samples were then incubated with primary antibodies at 4 °C overnight. The following day, after a washing step, the samples were incubated with appropriate secondary antibodies for 2 h at room temperature. For DHE staining, the sections were incubated with reactive oxygen species assay kit for superoxide anion with DHE (S0064, Beyotime). Images were acquired using a Leica fluorescence microscope (Stellaris, Leica, Mannheim, Germany) equipped with a 60× objective. Subsequent analysis of fluorescence intensity and binary reconstruction was performed using ImageJ software. The antibodies used were as follows: rabbit anti-GFAP (1:500, OB-PRB005, Oasisbiofarm), rabbit anti-iba1 (1:500, OB-PRB029, Oasisbiofarm), rabbit anti-NeuN (1:500, 26975-1-AP, Proteintech), rabbit anti-Phospho-Histone γH2A.X (1:500, 83307-2-RR, Proteintech).

*TEM*

Hippocampal tissues and HT22 cells were fixed with 2.5% glutaraldehyde for 30 min at room temperature and subsequently stored at 4 °C. The fixed samples were then dehydrated through a graded ethanol series and permeabilized with pure propylene oxide. Following this, they were embedded in epoxy resin and polymerized for 24 h. Ultrathin sections (70 nm thickness) were prepared using a Leica UC7 ultramicrotome. These sections were doubly stained with uranyl acetate and lead citrate before being examined under a Hitachi HT7800 transmission electron microscope for image acquisition.

*RNA-seq analyses*

Total RNA was extracted from the hippocampus using TRIzol® Reagent according the manufacturer’s instructions (Magen). RNA samples were detected based on the A260/A280 absorbance ratio with a Nanodrop ND-2000 system (Thermo Scientific, USA), and the RIN of RNA was determined by an Agilent Bioanalyzer 4150 system (Agilent Technologies, CA, USA). Differential expression analysis was performed using the DESeq2, DEGs with |log2FC|>1 and Padj < 0.05 were considered to be significantly different expressed genes. The GO and KEGG enrichment analysis of differential genes can explain the functional enrichment of differential genes and clarify the differences between samples at the gene function level. We use dplyr software package for GO function enrichment and KEGG pathway enrichment analysis. When P<0.05, it is considered that the GO or KEGG function is significantly enriched. Conventional enrichment analysis based on hypergeometric distribution relies on significantly upregulated or downregulated genes, and is prone to omitting some genes that are not significantly differentially expressed but have important biological significance.

*Materials*

Hydroge tetrachloroaurate trihydrate (HAuCl_4_•3H_2_O), ammonium hydroxide solution (28 wt % NH_3_ in H_2_O), Potassium carbonate (K_2_CO_3_), Formaldehyde solution, Isopropyl alcohol, and Acetone were from Shanghai Sinopharm Chemical Reagents Co., Ltd. Tetraethyl orthosilicate (TEOS, 99%), 1-ethyl-3(3-(dimethylamino)propyl) carbodimide hydrochloride (EDC), hydroxysuccinimide (NHS), (3-aminopropyl) triethoxysilane (APTES, 99%) were purchased from Aladdin Reagent Co.Ltd. Hexadecyltrimethylammonium bromide (CTAB, 99%) and were purchased from Sigma-Aldrich. Rabies virus glycoprotein-29 (RVG) was purchased from ChinaPeptides CO., Ltd. TPP-PEG-COOH was purchased from RuixiBiological Technology Co., Ltd.

*Synthesis of Au&mSiO_2_ Janus nanoparticles*

The Au nanoparticles were synthesized according to a previously reported method with slight modifications. Briefly, an aqueous solution of HAuCl_4_•3H_2_O (3 mL, 25 mM) was added to an aqueous solution of K_2_CO_3_ (200 mL, 50 mg) under vigorous stirring. The mixture was stirred for 30 s at room temperature, followed by aging in the dark for 17 h. Subsequently, the formaldehyde solution (1.334 mL) was added to the mixture. The reaction proceeded with stirring for 1 h, yielding a deep red colloidal suspension. The Au nanoparticles were collected by centrifugation at 5000 rpm for 10 min. Finally, the Au nanoparticles were resuspended in 20 mL of deionized water.

The Au&mSiO_2_ asymmetric nanocomposites were prepared based on the anisotropic growth method: The Au&mSiO_2_ asymmetric nanocomposites were prepared based on the anisotropic growth method: 11 mg of CTAB was added to 5 mL of a solution above Au nanoparticles. After sonication for 30 min, 125 μL of ammonia was added under continuous stirring. After stirring for 30 min, 12.5 μL of tetraethyl orthosilicate (TEOS) was added to the reaction solution and the reaction was continued for 1 h at 50 ℃. The Au&mSiO_2_ were collected by centrifugation and washed several times with water. The resulting nanomaterials were refluxed in acetone at 80 °C for 48 h three times to remove the surfactant CTAB and then washed with acetone three times.

*Synthesis of RVG-Au&mSiO_2_-TPP-VB*

10 mg of the Au&mSiO_2_ Janus nanoparticles were added to a solution containing 50 mL of isopropanol, followed by 50 μL of (3-aminopropyl) triethoxysilane (APTES) was added to amino-functionalize the mesoporous silica shell, which was stirred for 12 h at 70 °C, collected by centrifugation, and then washed with isopropanol three times. Then the materials were subsequently dispersed in water. Then the modified nanomaterials were then dispersed in 10 mL of deionized water. Subsequently, 2 mg of the RVG peptide was introduced, and the mixture was incubated under continuous stirring for over 12 h to facilitate conjugation. Following the reaction, the final product was collected by centrifugation, washed thoroughly to remove unbound peptides, and redispersed in PBS (pH 6.5) for subsequent applications. Afterwards the 60 mg of 1-(3-dimethylaminopropyl)-3-ethylcarbodiimide hydrochloride (EDC) and 80 mg of N-hydroxysuccinimide (NHS) adding TPP-PEG-COOH (5 mg), the mixture was allowed to react for 1 h at room temperature. Then, the solution of the RVG-Au&mSiO_2_-NH_2_ was added to the activated TPP solution. After stirring the reaction for 12 h, the RVG-Au&mSiO_2_-TPP nanocomposites were obtained by centrifugation and then washed three times with deionized water. 5 mg of VB was added to 10 mL of a solution above RVG-Au&mSiO_2_-TPP nanocomposites, which was stirred for 24 h at room temperature, and centrifugation at 7000 rpm for 10 min. Finally, the products were resuspended in 10 mL of deionized water. The remaining RES molecules were washed away with water. The loading of VB molecules was determined using UV based on their absorption peak area at 450 nm.

*Nanoparticles targeting detection*

HT22 cells were seeded in confocal dishes at a density of 1 × 10^5^ cells per well. After cell adhesion and upon reaching 60-70% confluency, the cells were co-cultured for 3 h with different types of nanoparticles (Au&mSiO_2_, RVG-Au&mSiO_2_, Au&mSiO_2_-TPP, RVG-Au&mSiO_2_-TPP, RVG-Au&mSiO_2_-TPP-VB) pre-labeled with PKH-26 dye (MINI26-1KT, Sigma-Aldrich). Following nanoparticle incubation, the culture medium was carefully removed, and mitochondria were stained with MitoBright LT Green dye (MT10, Dojindo), while nuclei were labeled with Hoechst dye (33342, Beyotime), by incubating at 37 °C for 20 min protected from light. After washing three times with PBS, fresh medium was added, and the samples were observed under a laser scanning confocal microscope.

Mice were nasally administered DiD-labeled (C1039, Beyotime), RVG-Au&mSiO_2_-TPP-VB nanoformulations. 12 h post-administration, the mice were euthanized, and brain tissues were collected for fixation, embedding, and cryosectioning. The sections were immunostained to label neurons (NeuN), microglia (IBA1), and astrocytes (GFAP), respectively. Colocalization of the nanoformulations with different neural cell types was examined using laser scanning confocal microscopy.

*In vivo imaging in small animals*

For in vivo tracking of the labeled agents, mice were anesthetized with isoflurane. The tracer, DiD-labeled RVG-Au&mSiO_2_-TPP-VB, was administered via intravenous or intranasal delivery at a dose of 50 mg kg^-1^. Following administration, whole-body and brain tissue imaging were performed at predetermined time points using the small animal live imaging system (AniView100). Fluorescence signals were captured using the appropriate filter sets (excitation/emission: 644 nm/663 nm). The acquired images were analyzed using AniView software to quantify the fluorescence intensity in regions of interest (ROIs).

*Endocytic uptake of PgO*

HT22 cells were seeded into confocal dishes at a density of 1 × 10^5^ cells per well and cultured in a standard incubator at 37 °C with 5% CO_2_ for 24 h to reach 60%-70% confluency. PgO was labeled with the fluorescent dye PKH-26 and co-cultured with HT22 cells for 1 h and 3 h. After fixation with 4% PFA, the nuclei and cytoskeleton were stained with DAPI (C1006, Beyotime) and Phalloidin (C2221, Beyotime), respectively. The phagocytosis of PgO by HT22 cells was observed under a laser scanning confocal microscope.

*Measurement of intracellular ROS and mitochondrial ROS*

Intracellular ROS levels were assessed in HT22 cells using the fluorescent probe DCFH-DA (S0033, Beyotime), following the manufacturer’s protocol as previously reported. In brief, cells were loaded with 10 μM DCFH-DA and incubated at 37 °C for 30 min. Fluorescence signals were captured by immunofluorescence microscopy at excitation/emission wavelengths of 488 nm and 525 nm, respectively.

Mitochondrial superoxide production was evaluated with the MitoBright ROS Deep Red Detection Kit (MT14, Dojindo). After staining, HT22 cells were imaged under a confocal microscope configured with excitation at 670 nm and emission at 540 nm. Quantitative analysis of fluorescence intensities for both DCFH-DA and MitoSOX Deep Red was performed using ImageJ software.

*Gene silencing approach*

To investigate the role of HIF-1α, siRNA-mediated knockdown was performed in HT22 cells. siRNA targeting mouse HIF-1α (HIF-1α_siRNA_; target sequence: 5’-GGAACAUGAUGGUUCACTT-3’) and a negative control siRNA were designed and synthesized by Sangon Biotech. Cells were transfected at 40-50% confluency using an appropriate transfection reagent. After 4 h of transfection, subsequent experimental treatments were initiated. Protein expression levels were assessed 48 h post-transfection to verify knockdown efficiency. In selected experimental groups, cells were treated with the NLRP3 activator nigericin (IN1880, Solarbio) (10 μM) following HIF-1α silencing. This combined approach enabled evaluation of pyroptosis-related signaling molecules and inflammatory factors in HT22 cells subjected to IH and/or PgO exposure.

*Western blot*

Following lysis, the tissue and cell samples were subjected to sonication and centrifugation. The resulting supernatant was collected, denatured, and separated via SDS-PAGE, after which the proteins were transferred to a PVDF membrane. The membrane was first blocked and then probed with primary antibodies, incubating at 4 °C overnight. After thorough washing, it was incubated with a secondary antibody for 1 h at room temperature. Finally, the target protein bands were detected using an enhanced chemiluminescence (ECL) system and quantified. The antibodies used for western blot are as follows: mouse anti-HIF-1 alpha (1:200, ab1, Abcam), rabbit anti-NLRP3 (1:1000, 15101, CST), mouse anti-TLR4 (1:1000, 19811-1-AP, Proteintech), rabbit anti-cleaved Gasdermin D (1:1000, 10137, CST), rabbit anti-cleaved caspase-1 (1:1000, 89332, CST), rabbit anti-β-actin (1:3000, GB15003-100, Servicebio).

*RT-qPCR*

Total RNA was subsequently isolated from the resulting lysates, as well as from tissues and cells, using Trizol reagent (Invitrogen, USA). The extracted RNA was then reverse-transcribed into complementary DNA (cDNA) with the Fasting PT Kit (TIANGEN, China). Quantitative reverse transcription PCR (RT-qPCR) analyses were conducted on a LightCycler® 96 system (Roche, Switzerland) employing the qPCR SYBR Green Master Mix (Yeasen, China). The nucleotide sequences of all primers used in this study are listed below.

Mouse-HIF1α-Forward	GACAAATCACCACAGGACA

Mouse-HIF1α-Reverse	GGGAGGAAATCAAGTCGTGC

Mouse-NLRP3-Forward	CCAAACCTCCAAGACCACTACG

Mouse-NLRP3-Reverse	CAGGGAAGAGATGCTCCTCAATGC

Mouse-TLR4-Forward	AGTACTATGTGATGTGACCATTGATG

Mouse-TLR4-Reverse	GAGACACCTGCCAGAGACATTGC

actin-Forward	CTGGAACGGTGAAGGTGACA

actin-Reverse	AAGGGACTTCCTGTAACAATG

*ELISA*

Hippocampal homogenates and serum samples were collected from mice, followed by centrifugation to obtain the supernatant for assay loading. A volume of 50 μL of standard/sample diluent was added to the blank wells, while 50 μL of standards or test samples were dispensed into the remaining wells. The plate was sealed and incubated at 37 °C for 40 min. After washing, the plate was blotted dry with filter paper. The primary antibody was added, the plate was resealed, and another wash cycle was performed. SABC reagent was then applied, and the plate was incubated at 37 °C for 30 min. Following subsequent washing, the chromogenic substrate was added. The reaction was terminated by adding 100 μL of stop solution to each well, and the absorbance was immediately measured at 450 nm using a microplate reader.

*Statistical analysis*

All bar chart data are expressed as mean ± standard deviation (SD), whereas violin plot data are presented as median and interquartile range (IQR). Quantification of fluorescence intensity was performed using ImageJ software, and statistical analyses were completed with GraphPad Prism 9.5 software. The normality of all data was verified via the Kolmogorov-Smirnov test, and the homogeneity of variances was assessed using the Levene’s test. Comparisons between two groups were conducted with the two-tailed unpaired t-test, while comparisons among multiple groups were performed using one-way analysis of variance (ANOVA), where appropriate. Statistical significance was defined as */#/△ *P* < 0.05; **/##/△△ *P* < 0.01; and ***/###/△△△ *P* < 0.001.
